# Comparative plasma metabolomics of Delta and Omicron SARS-CoV-2 variants: insights into variant-specific pathogenesis and therapeutic implications

**DOI:** 10.3389/fcimb.2025.1649724

**Published:** 2025-10-16

**Authors:** Eric Pimentel, Mohammad Mehdi Banoei, Chel Hee Lee, Brent W. Winston

**Affiliations:** ^1^ Department of Critical Care, Cumming School of Medicine, University of Calgary, Calgary, AB, Canada; ^2^ Department of Biomedical Engineering, Schulich School of Engineering, University of Calgary, Calgary, AB, Canada; ^3^ Department of Mathematics and Statistics, Faculty of Science, University of Calgary, Calgary, AB, Canada; ^4^ Departments of Medicine and Biochemistry and Molecular Biology, Cumming School of Medicine, University of Calgary, Calgary, AB, Canada

**Keywords:** COVID-19, SARS-CoV-2, Delta, Omicron, metabolomics, severity, treatment

## Abstract

**Background:**

The emergence of SARS-CoV-2 led to a global pandemic. Delta and Omicron, classified as concerning variants, differ significantly in transmissibility, disease severity, and antibody neutralization. Delta is associated with more severe disease, whereas Omicron is linked to increased transmissibility yet milder disease. This study investigates plasma metabolomic differences between Delta and Omicron infections and their associations with disease severity and treatment response. Importantly, this work examines variant-specific treatment metabolic effects – an aspect that remains underexplored despite the ongoing evolution of SARS-CoV-2 variants – and thus begins to fill a critical gap in the literature.

**Methods:**

A total of 109 hospitalized SARS-CoV-2 patients, confirmed by RT-PCR positivity (53 Delta, 56 Omicron), were matched by age and sex. Plasma samples collected on hospitalization days 1, 2, and 7 were analyzed using DI/LC-MS/MS-based (direct injection, liquid chromatography-tandem mass-spectrometry) targeted metabolomics. We employed univariate and multivariate statistical and pathway analyses to investigate and characterize metabolomic differences.

**Results:**

Distinct metabolic profiles differentiated Delta and Omicron infections. Specific metabolites, including tyrosine, asparagine, leucine, and acylcarnitines (C3, C4, C5), significantly distinguished variants and severity groups. Delta infections showed higher associations with severe outcomes. Corticosteroid treatment influenced metabolic profiles, revealing associations with modulation of metabolic and clinical responses.

**Conclusion:**

This study reveals significant plasma-based metabolic differences between Delta and Omicron SARS-CoV-2 variants, potentially reflecting their distinct clinical outcomes and severities.

## Introduction

Since the start of the COVID-19 pandemic, the Betacoronavirus pandemicum species Severe Acute Respiratory Syndrome-related Coronavirus-2 (SARS-CoV-2; family Coronaviridae, genus Betacoronavirus) has evolved, mutating into multiple variants of concern (VOC) with distinct transmissibility and severity of disease profiles (CDC, 2024). Two of the most clinically relevant VOC are Delta (lineage B.1.617.2) (Gomari et al., 2023) and Omicron (lineage B.1.1.529) (Dhawan et al., 2022), which emerged at different times and spread rapidly worldwide (Chan, 2022). Delta, identified in India in April 2021, demonstrated higher disease severity and hospitalizations, whereas Omicron, discovered in South Africa in November 2021, showed increased transmissibility but lower severity (Chan, 2022; Chavda et al., 2022). Data from Canada reflects these observations, with Delta resulting in a greater proportion of mechanically ventilated patients among hospitalized patients and Omicron producing a higher absolute case count, yet fewer mechanically ventilated cases in proportion to total hospitalizations (Canada Go, [[NoYear]]).

Several factors influence clinical outcomes of these variants, including mutations in the spike protein. Treatment and vaccination status also significantly influence clinical trajectories (Abdool Karim de Oliveira, 2021; Chan, 2022). Corticosteroids (e.g., dexamethasone) reduce hyperinflammatory responses and mortality in severe to critical COVID-19 cases (News, 2020), while vaccines significantly decrease COVID-19-related severity outcomes (Canada. Go, [[NoYear]]; Canada Go, [[NoYear]]; Canada IPaC, [[NoYear]]). Despite these benefits, the timing and nature of interventions, especially during different variant waves, may lead to distinct metabolic effects that remain poorly characterized but may help to understand the differential clinical responses seen in these variants. Because metabolomics captures the host biochemical state, the contrasting clinical phenotypes of Delta and Omicron provide a clear rationale to test whether these variants produce distinct plasma metabolomic signatures.

Metabolomics investigates small molecules (<1000 Da) in biofluids or tissues, providing insights into COVID-19 pathophysiology (Wishart, 2019). Key findings include disruptions in amino acid metabolism (particularly the tryptophan-kynurenine pathway), lipid metabolism and energy metabolism, linked to immune responses and disease progression (Albóniga et al., 2022; Liu et al., 2022; Lodge et al., 2023). Several metabolic biomarkers have been identified for diagnosis and prognosis. For instance, cytosine and AMP have been proposed as diagnostic biomarkers, while hexosylceramides and the arginine/kynurenine ratio have been associated with prognostic outcomes (Blasco et al., 2020; Fraser et al., 2020). Additionally, metabolic changes correlate with multi-organ dysfunction, particularly liver and kidney alterations (Cornillet et al., 2022; Gama-Almeida et al., 2023).

Metabolomics has contributed to predictive models and therapeutic strategies, such as modulation of the kynurenine pathway, and highlights sex-specific metabolic variations (Fraser et al., 2020; Ghini et al., 2023; Lima et al., 2024). By leveraging techniques like liquid chromatography-mass spectrometry (LC-MS/MS) (Xiao et al., 2012) and direct injection–mass spectrometry (DI-MS/MS) (Boccard et al., 2010), researchers can capture the biochemical status of patients at a point in time. Prior work has shown that severe COVID-19 is often associated with elevated phenylalanine, kynurenine, and glucose (Rahnavard et al., 2022), suggesting hyperinflammatory and energy-intensive processes. Moreover, SARS-CoV-2 infection may cause long-lasting metabolic perturbations, as seen in cases of long COVID (Blasco et al., 2020; Fraser et al., 2020).

Importantly, as SARS-CoV-2 continues to evolve, most recently with the NB1.8.1 lineage under close monitoring – there is an urgent need for variant-specific therapeutic metabolomics data. Few studies to date have integrated metabolomic insights directly into treatment stratification across evolving VOCs.

Within this framework, we aimed to investigate whether the differences in transmissibility and severity of Delta and Omicron SARS-CoV-2 variants manifest distinct plasma metabolomic profiles, potentially revealing pathways underlying disease mechanisms. Furthermore, we sought to determine whether corticosteroid therapy and vaccination modify the plasma metabolic signatures of Delta and Omicron infections, and whether these metabolic effects help explain the differential clinical responses.

## Materials and methods

### Study ethics approval and patient selection

This study was approved by the Conjoint Health Research Ethics Board (CHREB) at the University of Calgary (Ethics ID: REB23-0457_RENI). Additionally, ethics approval for the collection and use of plasma samples from the Biobanque Québécoise de la COVID-19 (BQC-19) was reviewed and approved by the Research Ethics Board of the Center Hospitalier de l’Université de Montréal (REB-CHUM). The BQC-19, a decentralized biobank managed by McGill University and the Research Institute of the McGill University Health Centre (RI-MUHC), contains data and biological specimens from approximately 3,000 COVID-19 patients in Quebec, Canada. Plasma samples from 109 hospitalized patients were included in this study, with 53 infected with the SARS-CoV-2 Delta variant and 56 with the Omicron variant, manually matched by age (± 5 years) and sex using an Excel spreadsheet.

Inclusion criteria required patients to (1): be hospitalized with confirmed COVID-19 infection via real-time polymerase chain reaction (RT-PCR) (2), be aged ≥18 years (3), provide consent to participate in the biobank and related studies, and (4) be categorized by severity groups using the NIH classification criteria, as well as vaccination status following the criteria established by the Government of Canada for complete and incomplete vaccination (Canada Go, 2023). Vaccine product/brand was not uniformly available across participants; therefore, vaccination was analyzed as completeness (≥2 vs. <2 doses). Among the subset with product information, most received mRNA vaccines—Pfizer-BioNTech (n=20) or Moderna (n=6)—with fewer adenoviral-vector (AstraZeneca/Covishield; n=2) or protein-subunit (Novavax; n=1) products. Exclusion criteria included (1): patients testing negative by RT-PCR (2), patients or surrogates unwilling to participate in the biobank and studies (3), patients <18 years old, and (4) patients who did not have a plasma sample from day 1 and at least one from either day 2 or day 7.

### Sample collection

Blood samples were collected in acid citrate dextrose (ACD) tubes, then centrifuged at 850×g for 10 minutes at room temperature. Plasma was transferred into 500 µL and 250 µL aliquots and stored at −80°C. Samples were drawn on days 1, 2, and 7 of hospitalization, resulting in a total of 302 plasma samples (144 Delta, 158 Omicron) used in this study. Of these, 109 were collected on day 1 (53 Delta, 56 Omicron), 103 on day 2 (52 Delta, 51 Omicron), and 90 on day 7 (39 Delta, 51 Omicron). Aliquots were shipped on dry ice to the Critical Care Epidemiologic and Biologic Tissues Resource (CCEPTR, a tissue bank) at the University of Calgary for management and further processing.

### Sample preparation for metabolomics analysis

All samples were stored at −80°C immediately upon arrival at the University of Calgary. At the University of Calgary, the samples were thawed on ice, and 100 µL aliquots were re-labeled, refrozen at -80°C, and then shipped on dry ice for metabolite quantification. All sample management followed biosafety level 2 protocols.

For organic acid, amino acid and lipid quantifications, procedures are provided in the Methods section of the **Supplementary Material**.

### Mass spectrometry analysis

A total of 143 metabolites were measured using LC-MS/MS and DI-MS/MS (**Supplementary Table S1**).

For mass spectrometry analysis and data processing details, see the Methods section of the **Supplementary Material**.

### Statistical analyses

Initially, a univariate analysis for each metabolomic and demographic variable was conducted. For continuous variables, the data were summarized using the mean and standard deviation. Group comparisons between two categories were performed using the Wilcoxon rank-sum test. For categorical variables, frequencies and percentages were presented, and group differences were assessed using either the chi-square or Fisher’s exact test, selected based on cell counts. Statistical significance was determined at a p-value of less than 0.05. Furthermore, a two-way analysis of variance (ANOVA) was employed to understand the main and interaction effects of the two factors.

Subsequently, multivariate analyses were conducted. Principal component analysis (PCA) was used to identify potential outliers and inherent data structures. For exploratory data visualization, a heatmap was created, and hierarchical clustering was carried out using Euclidean distance as the similarity metric and Ward’s linkage for cluster aggregation. To clarify group discrimination and identify key metabolites, partial least squares discriminant analysis (PLS-DA) was employed. The R2 and Q2 statistics were applied to evaluate the model’s explanatory and predictive powers, respectively. Finally, pathway analysis was performed using over-representation analysis. All statistical procedures were carried out using MetaboAnalyst 6.0.

## Results

### Clinical and demographic characteristics

A total of 109 patients were enrolled in this study, comprising 53 individuals infected with the Delta variant and 56 with the Omicron variant (**Table 1**). The median age was 61.3 years for Delta and 59.3 years for Omicron, with no statistically significant difference (p=0.794) in age. Similarly, the sex distribution was alike between the two groups because they were matched by sex (66% male in Delta vs. 67.9% in Omicron; p=1.00). This match was purposefully designed to reduce confounding metabolomic variability, given the influence of age and sex on metabolites (Costanzo et al., 2022).

**Table 1:** Patient’s demographics.

Characteristics	Category	Overall (n=109)	Delta (n=53)	Omicron (n=56)	P-value*	Missing (%)
Age (median[IQR])		59.70 [46.50, 71.30]	61.30 [45.10, 71.30]	59.30 [47.60, 69.30]	0.794	
Sex (%)	Female	36 (33.0)	18 (34.0)	18 (32.1)	1	
	Male	73 (67.0)	35 (66.0)	38 (67.9)		
Days in hospital		17 [10.50, 24.50]	15 [9.00, 22.00]	20 [13.25, 31.00]	0.013	2 (1.8)
(median [IQR])						
ICU (%)	Non-ICU	55 (51.4)	28 (52.8)	27 (50)	0.921	2 (1.8)
	ICU	52 (48.6)	25 (47.2)	27 (50)		
NIH-based severity (%)	Mild	22 (20.2)	6 (11.3)	16 (28.6)	0.001	
	Moderate	21 (19.3)	9 (17.0)	12 (21.4)		
	Severe	41 (37.6)	30 (56.6)	11 (19.6)		
	Critica	25 (22.9)	8 (15.1)	17(30.4)		
Severity (%)	mild+mod	43 (39.4)	15 (28.3)	28 (50.0)	0.034	
	sev+crit	66 (60.6)	38 (71.7)	28 (50.0)		
Vaccination (%)	<2 vaccines	17 (27.9)	13/31 (41.9)	4/30 (13.3)	0.042	48 (43.1)
	≥2 vaccines	44 (72.1)	18/31 (58.1)	26/30 (86.7)		
Systemic corticosteroid use (%)	Not administered	30 (27.5%)	11 (20.8)	19 (33.9)	0.185	
	Administered	79 (72.5%)	42 (79.2)	37 (66.1)		
Remdesivir use (%)	Not administered	85 (78.0)	42 (79.2)	43 (76.8)	0.937	
	Administered	24 (22.0)	11 (20.8)	13 (23.2)		
Tocilizumab (%)	Not administered	85 (78.0)	33 (62.3)	52 (92.9)	<0.001	
	Administered	24 (22.0)	20 (37.7)	4 (7.1)		
Rivabirin (%)	Not administered	109 (100.0)	53 (100.0)	56 (100.0)	NA	
	Administered	0 (0.0)	0 (0.0)	0 (0.0)		
Lopinavir (%)	Not administered	109 (100.0)	53 (100.0)	56 (100.0)	NA	
	Administered	0 (0.0)	0 (0.0)	0 (0.0)		

*p-value obtained from Chi-square test of independence and Fisher’s exact test when frequencies were less than 5. NA, not appropriate

Hospital stay duration differed significantly: the Omicron group had a longer median stay (20 days; IQR = 13.25-31) than the Delta group (15 days; IQR = 9.00–22.00, p = 0.013). Although ICU admission rates were similar (47.2% Delta vs 50% Omicron, p = 0.921), NIH-based severity classification revealed that Delta patients had a greater proportion of severe and critical cases combined (71.7%) compared to Omicron (50.0%, p=0.133). Conversely, Omicron exhibited a higher rate of mild and moderate cases (50.0%) compared to Delta patients (28.3%, p=0.033). Complete vaccination (≥2 doses) was more common in Omicron cases (86.7%) than in Delta (58.1%, p = 0.145), whereas fewer than two doses were observed in 41.9% of Delta versus 13.3% of Omicron patients (p = 0.026). Corticosteroid usage rates were high overall (79.2% Delta vs. 66.1% Omicron, p=0.326), while tocilizumab administration differed significantly (37.7% in Delta vs. 7.1% in Omicron, p=0.001) (**Table 1**).

### Plasma metabolomic differences between Delta and Omicron patients

Twenty-two metabolites were significantly altered (p<0.05) in the plasma of Delta-infected patients compared to those infected with Omicron. Among these, metabolites with increased median concentrations in Delta relative to Omicron included asparagine, asymmetric dimethylarginine, decanoylcarnitine (C10), propionylcarnitine (C3), butyrylcarnitine (C4), hydroxybutyrylcarnitine (C4OH), valerylcarnitine (C5), glutamine, isoleucine, leucine, lysophosphatidylcholine acyl (LysoPC a) C18:2, LysoPC a C20:3, LysoPC a C20:4, phosphatidylcholine diacyl (PC aa) C38:6, PC aa C40:6, threonine, and tyrosine. Conversely, aspartic acid, octadecenoylcarnitine (C18:1), homocysteine, and taurine exhibited decreased median concentrations in Delta relative to Omicron (see **Supplementary Table S1.1** for day-specific fold changes). For an overview of all the metabolites significantly altered between variants, refer to **Supplementary Figure S1A**.

**Figure 1:**
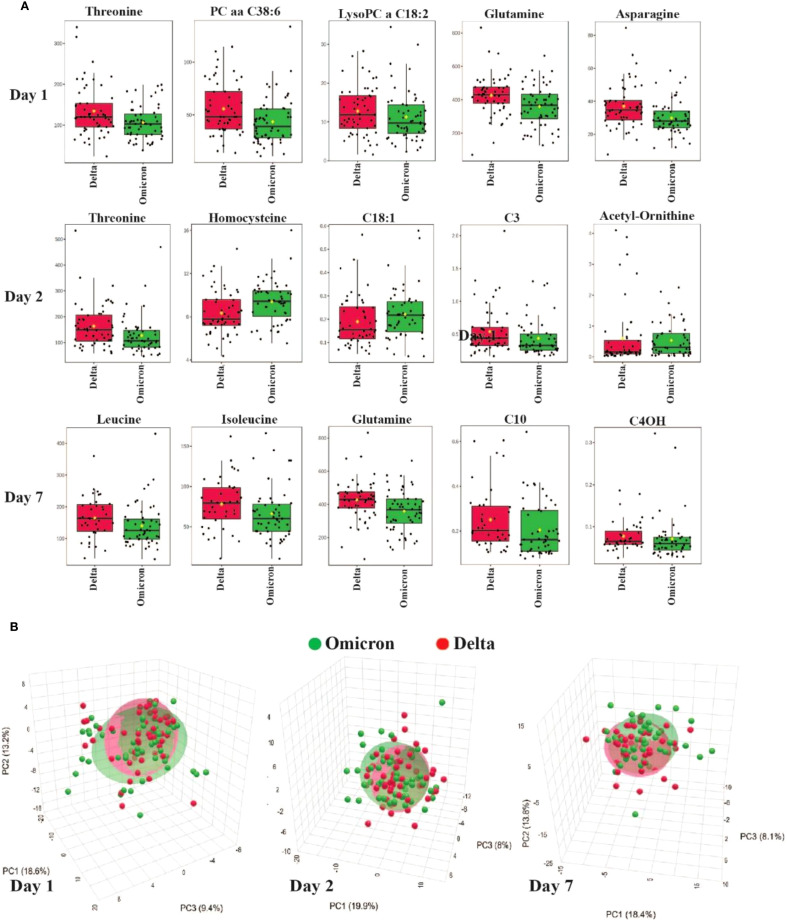
Metabolomic differences between Delta and Omicron SARS-CoV-2 variants. **(A)** Boxplots showing the most significantly altered plasma metabolites differentiating Delta and Omicron variants on days 1, 2 and 7 of hospitalization. **(B)** PCA illustrating metabolic clustering of Delta and Omicron variants on days 1, 2 and 7. There is considerable overlap of the two variants, but there are also visible differences.

**Figure 1** provides a visual representation of these metabolomic differences between Delta and Omicron-infected patient plasma metabolites. Panel A shows the boxplots of the most significantly altered metabolites between the Delta and Omicron variants on days 1, 2 and 7 of hospitalization, emphasizing the variant differences. Panel B is a principal component analysis (PCA) showing the metabolic clustering between the groups, in Delta and Omicron patient plasma. Overall, as expected, there is a great deal of overlap between the cohorts, but there are visible differences between them. There are also inherent differences within the cohorts, which are likely due to the inherent heterogeneity within the patient cohorts, including factors such as disease severity, comorbidities, and metabolic responses.

Pathway analysis incorporated all metabolites that showed a significant difference between the Delta and Omicron variants on days 1, 2 and 7 (**Supplementary Figure S2A**; **Supplementary Table S2**). The top pathways with the greatest impact scores related to these variant differences, in descending order, were phenylalanine, tyrosine, and tryptophan biosynthesis; taurine and hypotaurine metabolism; alanine, aspartate, and glutamate metabolism; tyrosine metabolism; and cysteine and methionine metabolism. Whereas, the most statistically significant pathways, listed in descending order, were valine, leucine, and isoleucine biosynthesis; arginine biosynthesis; alanine, aspartate, and glutamate metabolism; valine, leucine, and isoleucine degradation; and phenylalanine, tyrosine, and tryptophan biosynthesis pathways.

### Severity-based metabolite differences

Thirty-two metabolites were significantly altered (p<0.05) in the plasma of the severe and critical group (SC) compared to the mild and moderate (mM) group. Metabolites with increased median concentrations in SC group included alanine, alpha-aminoadipic acid, asparagine, carnitine (C0), C3, C4, C5, creatine, fumaric acid, glutamine, homovallinic acid, kynurenine, lactic acid, leucine, lysine, LysoPC a C20:4, methionine, methionine-sulfoxide, ornithine, PC aa C32:2, phenylalanine, pyruvic acid, succinic acid, threonine, tryptophan, tyrosine, and valine. Conversely, acetyl-ornithine, homocysteine, and trans-hydroxyproline exhibited decreased median concentrations in the SC group compared to the mM group. For an overview of all metabolites significantly altered between severity groups, refer to **Supplementary Figure S1B**.

**Figure 2:**
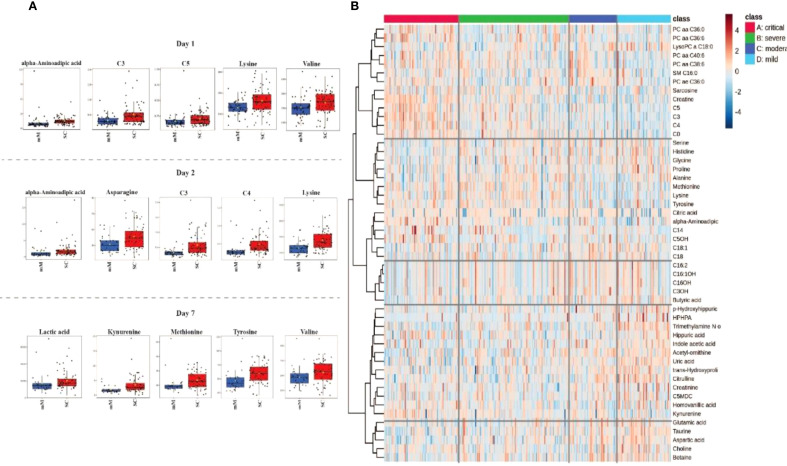
Metabolomic profiling associated with COVID-19 severity in Delta and Omicron infected individuals. **(A)** Boxplots representing the most significant metabolite changes differentiating mild/moderate (mM) from severe/critical (SC) cases on days 1, 2 and 7 of hospitalization. **(B)** Heatmap illustrating the top 50 metabolites distinguishing between critical, severe, moderate, and mild cases in all days of hosptilization (1, 2 and 7) characterizing their distinct metabolic signatures.

**Figure 2** provides a comprehensive visualization of these severity-based metabolomic differences. Panel A displays boxplots of the most significantly altered metabolites between mild/moderate (mM) and severe/critical (SC) cases on days 1,2 and 7 of hospitalization, emphasizing their potential role in disease progression. Panel B presents a heatmap displaying the metabolic profiles across COVID-19 severity groups (critical, severe, moderate, mild), highlighting significant variations in metabolite levels associated with disease severity. Critical cases (marked in red) exhibit a unique metabolic signature compared to less severe cases, reflecting profound metabolic disruptions linked to critical illness. The hierarchical clustering analysis groups metabolites with similar expression patterns, revealing clusters of lipid metabolites (e.g., PC aa C36:0, LysoPC C18:0) that are predominantly altered in severe and critical cases. Additionally, amino acids and intermediates related to energy metabolism (e.g., kynurenine, methionine, tyrosine) are distinctly elevated in critically ill patients, indicating metabolic stress and dysregulation of amino acid metabolism. The clustering patterns emphasize co-regulated metabolites that may play critical roles in the pathophysiology of severe COVID-19 and serve as potential biomarkers for disease severity.

**Figure 3:**
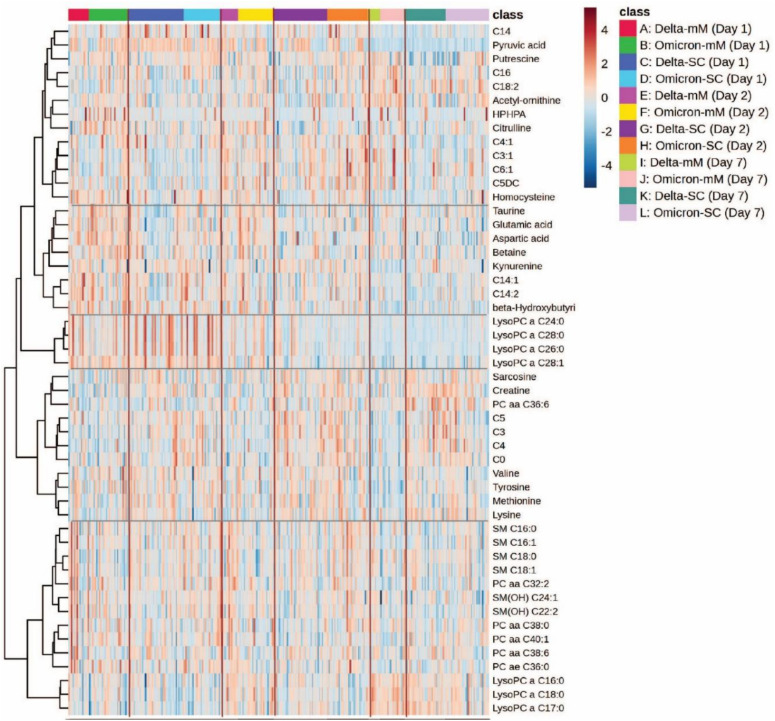
Heatmap analysis representing hierarchical clustering of plasma metabolites from COVID-19 patients infected with Delta and Omicron variants. The heatmap illustrates metabolomic profiles across three time points (day 1, day 2 and day 7). The x-axis represents individual patient samples, categorized by variant type (Delta or Omicron), disease severity (mild/moderate [mM] or severe/critical [SC]), and sampling day.

**Figure 3** illustrates distinct metabolic signatures associated with SARS-CoV-2 variants (Delta and Omicron) and disease severity (mild to severe) over three time points (Days 1, 2 and 7). Critical cases exhibit substantial upregulation of lipids (e.g., LysoPC and SM species) and amino acids (e.g., lysine, methionine, tyrosine) compared to milder cases. Metabolite clustering reveals coordinated regulation patterns, highlighting potential biomarkers that differentiate variant types and disease severity, reflecting the dynamic metabolic alterations over time.

Furthermore, the metabolic interaction effects of severity (m+M and S+C) and SARS-CoV-2 variants (Delta and Omicron) were assessed. A comprehensive summary of the two-way ANOVA results for days 1, 2 and 7 are provided in **Supplementary Table 3**. Fifteen metabolites demonstrated significant interaction effects, Pr (>F) 3, p <0.05, including asymmetric dimethylarginine, betaine, creatine, creatinine, fumaric acid, isoleucine, leucine, lysine, LysoPC C18:0, methylhistidine, PC aa C38:0, spermidine, spermine, total dimethylarginine, and valine (**Supplementary Figure S3**).

Finally, pathway analysis included all significantly altered metabolites associated with COVID-19 severity between the Delta and Omicron variants on days 1, 2 and 7 (**Supplementary Figure S2B**; **Supplementary Table S2**). The pathways with the greatest impact on severity, in descending order, were phenylalanine, tyrosine, and tryptophan biosynthesis; alanine, aspartate, and glutamate metabolism; phenylalanine metabolism; cysteine and methionine metabolism; and tryptophan metabolism. In contrast, the most statistically significant pathways, also listed in descending order, were alanine, aspartate, and glutamate metabolism; arginine biosynthesis; valine, leucine, and isoleucine biosynthesis; citrate cycle (TCA cycle); and phenylalanine, tyrosine, and tryptophan biosynthesis.

### Effects of corticosteroid treatment on plasma metabolites in Delta and Omicron-infected individuals

A comprehensive summary of the metabolite profiles affected by corticosteroid treatment, categorized as non-corticosteroid use and corticosteroid use on days 1, 2 and 7 of hospitalization, is provided in **Supplementary Figure 1C**. **Supplementary Figure S1C** illustrates the significant median changes in metabolomic concentrations associated with corticosteroid use.

Twenty-six metabolites were significantly altered (p<0.05) in the plasma of the corticosteroid use group compared to the non-corticosteroid use group. Metabolites with increased median concentrations in the treated group included alanine, alpha-aminoadipidic acid, asparagine, beta-hydroxybutyric acid, C3, C4, C5, fumaric acid, glutamine, leucine, lysine, methionine, PC aa C32:2, phenylalanine, SM (OH)C14:1, SM(OH)C22:1, threonine, tryptophan, tyrosine, and valine. In contrast, acetyl-ornithine, asymmetric dimethylarginine, indole acetic acid, LysoPC a C18:0, and total dimethylarginine showed decreased median concentrations in the corticosteroid use group compared to the non-corticosteroid use group.

**Figure 4:**
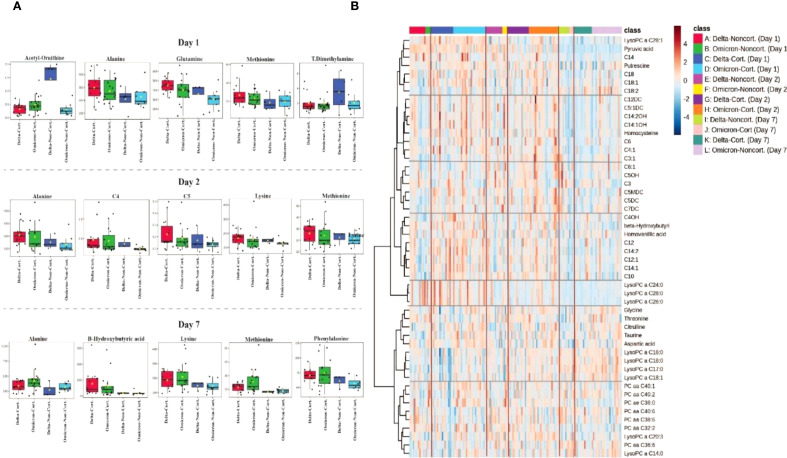
Metabolomic differences associated with corticosteroid treatment in COVID-19 patients. **(A)** Boxplots depicting the most significant plasma metabolite alterations between corticosteroid-treated (Cort.) and non-treated (Non-cort.) patients at days 1, 2 and 7 of hospitalization, stratified by SARS-CoV-2 variant (Delta and Omicron). **(B)** Heatmap analysis illustrating hierarchical clustering and metabolic signatures of the top 40 metabolites differentiating patients treated with corticosteroids from those without corticosteroid treatment across the three sampling days.

In **Figure 4B**, a heatmap illustrates the hierarchical clustering of metabolites based on their abundance in COVID-19 patients receiving corticosteroids and those who did not receive corticosteroids at three time points (Days 1, 2 and 7) of the study for both Delta and Omicron variants. The color gradient ranges from blue to red, indicating low and high metabolite abundance, respectively. Clustering patterns reveal metabolic differences linked to corticosteroid treatment and variant type. Patients who received corticosteroids show a marked increase in certain metabolites, such as lipids (e.g., LysoPC and SM species) and amino acids (e.g., lysine and methionine), compared to patients who did not receive corticosteroids, implying a significant metabolic effect of corticosteroid therapy. Furthermore, alterations in metabolic pathways influenced by treatment and variant type result in clusters of coordinated metabolites. These findings suggest that corticosteroid administration may elicit different metabolic responses in SARS-CoV-2 variants and could serve as potential therapeutic and prognostic biomarkers.

Moreover, the metabolic interaction effects of corticosteroid use (non-corticosteroid use and corticosteroid use) and SARS-CoV-2 variant (Delta and Omicron) were evaluated. A comprehensive summary of the two-way ANOVA results from days 1, 2 and 7 is provided in **Supplementary Table 4**. Fourteen metabolites demonstrated significant interaction effects, Pr (>F) 3, p-value <0.05 which included: C0, C4, glycine, homocysteine, indole acetic acid, kynurenine, LysoPC a C14:0, LysoPC a C18:1, PC aaC36:0, PC aa C40:1, phenylalanine, hydroxyhippuric acid, hydroxysphingomyeline C24:1, and taurine (**Supplementary Figure S4**).

Pathway analysis incorporates all significant metabolites associated with corticosteroid treatment, comparing Delta and Omicron variants on days 1, 2 and 7 (**Supplementary Figure S2C**; **Supplementary Table S2**). The pathways exhibiting the greatest impact, in descending order, included phenylalanine, tyrosine, and tryptophan biosynthesis; phenylalanine metabolism; tyrosine metabolism; alanine, aspartate, and glutamate metabolism; and tryptophan metabolism. In contrast, the most statistically significant pathways, also listed in descending order, were alanine, aspartate, and glutamate metabolism; arginine biosynthesis; valine, leucine, and isoleucine biosynthesis; phenylalanine, tyrosine, and tryptophan biosynthesis; and phenylalanine metabolism.

### Effects of vaccination on plasma metabolites in Delta and Omicron infection

A comprehensive summary of the metabolite profiles affected by vaccination, categorized as incomplete and complete vaccination, on days 1, 2 and 7 of hospitalization, is provided in **Supplementary Figure 1D**. **Supplementary Figure S1D** illustrates the significant median changes in metabolomic concentrations associated with vaccination.

Twenty-three metabolites were significantly altered (p<0.05) in the plasma of the completely vaccinated group compared to the incompletely vaccinated group. Metabolites with increased median concentrations in the completely vaccinated group included acetyl-ornithine, C10:1, C4OH, citrulline, glycine, isobutyric acid, propionic acid, taurine, hydroxyproline and uric acid. Conversely, alpha-aminoadipic acid, ketoglutaric acid, asparagine, C3, C4, lysine, LysoPC a C26:1, methionine, PC aa C36:6, proline, pyruvic acid, SM C20:2, and threonine showed decreased median concentrations in the completely vaccinated group compared to the incompletely vaccinated group.

**Figure 5:**
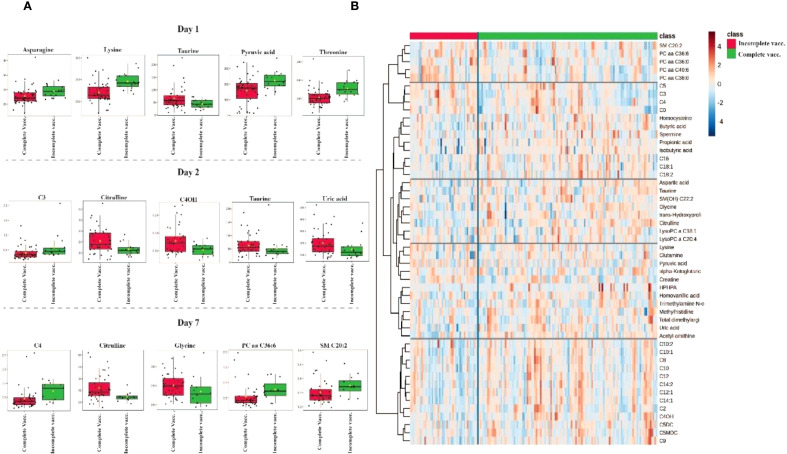
Metabolomic differences between completely vaccinated and incompletely vaccinated COVID-19 patients. **(A)** Boxplots of the most significantly altered metabolites between completely vaccinated and incompletely vaccinated groups on days 1, 2 and 7 of hospitalization. **(B)** Heatmap displaying hierarchical clustering of metabolite abundance between groups, with a color gradient representing relative metabolite levels (red = high, blue = low). Distinct clustering patterns suggest metabolic differences influenced by vaccination status.

**Figure 5** provides a comprehensive visualization of the metabolomic differences between vaccination groups. **Figure 5A** displays boxplots of the most significantly altered metabolites between completely vaccinated and incompletely vaccinated cases on days 1, 2 and 7 of hospitalization. **Figure 5B** depicts a heatmap of the top 40 metabolites, illustrating clustering patterns based on vaccination.

Furthermore, the metabolic interaction effects of vaccination status (complete vaccination and incomplete vaccination) and SARS-CoV-2 variant (Delta and Omicron) were evaluated. A comprehensive summary of the two-way ANOVA results from days 1, 2 and 7 are provided in **Supplementary Table 5**. Three metabolites demonstrated significant interaction effects, Pr (>F) 3, p-value <0.05, which included: taurine, C7DC, and C5DC (**Supplementary Figure S5**).

Finally, pathway analysis incorporates all significant metabolites associated with vaccination status, comparing Delta and Omicron variants on days 1, 2 and 7 (**Supplementary Figure S2D**, see **Supplementary Table S2**). The pathways exhibiting the greatest impact, in descending order, included glycine, serine, and threonine metabolism; arginine biosynthesis; alanine, aspartate and glutamate metabolism; arginine and proline metabolism; and lipoic acid metabolism. In contrast, the most statistically significant pathways, also listed in descending order, were arginine biosynthesis; alanine, aspartate, and glutamate metabolism; lipoic acid metabolism; glycine, serine and threonine metabolism; and arginine and proline metabolism.

## Discussion

Comparative analysis of the metabolomic profiles between the Delta and Omicron variants identified distinct metabolic changes associated with each variant. Specifically, the Delta variant led to increased concentrations of amino acids, ACs, and LysoPCs, whereas Omicron infection was characterized by elevated levels of aspartic acid, homocysteine, and taurine. Despite considerable overlap in metabolic signatures shared by both variants, pathway enrichment analysis indicated variant-specific biochemical perturbations, particularly within amino acid metabolism pathways.

Further, disease severity markedly impacted metabolism, with severe and critical COVID-19 cases exhibiting significant changes related to energy metabolism, immune dysregulation, and protein degradation (Caterino et al., 2021; Roberts et al., 2023). Increased concentrations of alanine, kynurenine, and branched-chain amino acids (BCAAs; leucine, isoleucine, and valine) suggest enhanced protein catabolism and altered immune function (Caterino et al., 2021). Corticosteroid therapy further modified metabolic profiles, elevating alanine, glutamine, and tryptophan while lowering acetylornithine. These observations suggest adaptive metabolic responses to immune modulation and altered energy metabolism (Caterino et al., 2021; Roberts et al., 2023), reinforcing the concept that host metabolic reactions are influenced by the viral variant, disease severity, therapeutic intervention, and individual host factors.

Clinically, we observed variations in hospitalization duration and disease severity between variants. Although Omicron-infected individuals had a longer median hospital stay, Delta cases presented a higher incidence of severe and critical outcomes. The increased severity in Delta-infected patients likely arises from the development of diffuse alveolar damage (DAD) or acute hypoxemic respiratory failure and lower complete vaccination rates, predisposing them to greater inflammatory responses and metabolic disruptions (Fall et al., 2022; Jeican et al., 2023). Conversely, the milder clinical presentations associated with Omicron infections—potentially reflecting higher complete vaccination rates and inherently reduced pathogenicity of the variant (Bálint et al., 2022) aligned with metabolomic profiles indicative of lower systemic inflammation and metabolic stress.

Our findings corroborate earlier metabolomic analyses of COVID-19, highlighting shared metabolic abnormalities during disease progression and viral infection. Consistent with previous research, our study underscores the significance of amino acid metabolism, energy metabolism, and lipid dysregulation in COVID-19 pathogenesis. Both our results and referenced studies identified elevated kynurenine levels in severe cases, confirming the involvement of tryptophan metabolism in immune modulation (Blasco et al., 2020). Additionally, altered BCAA concentrations suggest roles in protein degradation and immune function (Cruzat et al., 2018; Rahnavard et al., 2022).

Corticosteroid treatment, more frequently used in severe Delta cases due to intense inflammatory responses, significantly altered inflammatory-related metabolites compared to Omicron cases (van Paassen et al., 2020; Horby et al., 2021; Neufeldt et al., 2022). Corticosteroid administration produced distinct metabolic signatures, characterized by increased methionine, glutamine, phenylalanine, and alanine—indicators of protein catabolism and gluconeogenesis—and decreased acetylornithine levels, implying effects on the urea cycle and nitrogen balance (Chung et al., 2015; Williams, 2018; Imoto et al., 2022). These biochemical alterations reflect the catabolic state induced by corticosteroids, emphasizing the importance of tailoring therapeutic strategies to manage associated metabolic effects.

Metabolomic comparisons of SARS-CoV-2 variants further explored how specific metabolite alterations affect host immune functions and disease progression. The Delta variant prominently disturbed amino acid metabolism, particularly arginine, glutathione, and tryptophan pathways, contributing to inflammation and oxidative stress (Lee et al., 2024; Rajaiah et al., 2024). Delta has also been shown to promote hyperglycemia and altered glucose transporter expression, facilitating viral propagation (Rochowski et al., 2024).

Hyperglycemia and advanced glycation end-products (AGEs) enhanced viral entry and oxidative stress (Michaels et al., 2024). While Delta exhibits more severe metabolic effects, Omicron evades the immune system through different metabolic mechanisms (Chan, 2022). These findings indicate that variant-specific metabolic phenotypes can provide crucial biomarkers for COVID-19 severity assessment and therapeutic interventions.

Temporal analysis revealed distinct metabolic alterations corresponding to infection progression. Early (day 1) increases in asparagine, glutamine, LysoPCs, and threonine, particularly in Delta infections, indicate initial immune activation and inflammation, as glutamine supports lymphocyte proliferation and LysoPCs mediate inflammatory signaling (Cruzat et al., 2018; Qaradakhi et al., 2020; Tan et al., 2020; Li et al., 2021; Jin et al., 2023). By day 2, metabolic adaptations were evident through altered threonine, homocysteine, oleic acid (C18:1), and propionic acid (C3) levels, implicating ongoing protein synthesis, oxidative stress management, and immune regulation (Tang et al., 2021). Persistent alterations in acetylornithine underscore sustained metabolic stress and urea cycle disruption in SARS-CoV-2 infection.

By day 7, metabolite profiles further diverged, particularly in Delta cases, displaying significant elevations in metabolites such as C4, C4OH, PC ae C40:6, capric acid (C10), tyrosine, asymmetric dimethylarginine (ADMA), and valeric acid (C5). These changes likely reflect prolonged inflammatory responses and metabolic stress, potentially explaining worse clinical outcomes in Delta infections (Nie et al., 2018; Al-Mekhlafi et al., 2021; Jung et al., 2021; Pimentel et al., 2024). Pathway analyses underscored disruptions in alanine, aspartate, glutamate metabolism, phenylalanine, tyrosine, tryptophan biosynthesis, and taurine metabolism pathways, highlighting their roles in energy demand, neurotransmitter synthesis, immune modulation, and oxidative stress management (Cruzat et al., 2018; Qaradakhi et al., 2020; Tan et al., 2020; Li et al., 2021; Jin et al., 2023).

Despite these compelling insights, limitations warrant acknowledgment. Exclusion of certain critical metabolites due to targeted analysis and sample collection constraints (use of acid citrate dextrose (ACD) tubes for sample collection), limits examination of some metabolites. A predominantly male population (67%) and significant missing vaccination data (43.1%) constrain vaccine-related generalizability. Additionally, the absence of a SARS-CoV-2 PCR-negative control group precludes direct comparison with uninfected individuals. Further research involving larger and diverse cohorts is needed to validate these observations.

There are also additional limitations related to potential confounders (eg., diet), and selection bias inherent to a non-random, retrospective design. Comorbidity burden (e.g., diabetes, chronic kidney/liver, cardiovascular or chronic lung disease, obesity) may prolong recovery and length of stay (LOS). Baseline comorbidity data were unavailable, and the “condition” variables captured largely represent in-hospital events (some possibly present on admission). Because these conditions can influence the metabolome and may lie on the causal pathway, we treated them as outcomes rather than covariates to avoid over-adjustment. Accordingly, we report their between-variant differences as exploratory, unadjusted risk differences (**Supplementary Figure S6**) with full counts and 95% confidence intervals (CIs) (**Supplementary Table S6**), whereas our primary mortality inference is based on a covariate-adjusted Cox model using only baseline admission covariates (**Supplementary Table S7**). Another limitation is that patient-level viral-load data were unavailable, therefore some metabolite differences may reflect viral-load variation rather than purely variant effects.

Despite these limitations we believe this work contributes to the understanding of metabolomic signatures in Delta and Omicron infections. Further detail is provided in the Limitations **Supplementary Material**.

## Conclusion

In summary, this study shows that SARS-CoV-2 variants induce distinct clinical and metabolic responses. Delta infections were characterized by increased severity and prolonged metabolic stress compared to Omicron, reflecting variant-specific pathophysiological mechanisms. Importantly, the interplay between vaccination status and corticosteroid use emerged as an important factor influencing these metabolomic alterations. Higher vaccination rates in Omicron cases were associated with moderated metabolic profiles, while the therapeutic impact of corticosteroids varied considerably between variants. These findings not only deepen our understanding of the metabolic disruptions underlying COVID-19 severity but also show potential metabolic targets for therapeutic intervention. This work paves the way for tailored diagnostic and treatment strategies, showing the need for variant-specific approaches to optimize patient outcomes in the evolving landscape of ongoing COVID-19.

## Data Availability

The clinical data used in this study were obtained from the Biobanque Québécoise de la COVID-19 (BQC19) and are subject to third-party access restrictions. Researchers may request access directly from BQC19 via its data access portal: https://bqc19.bento.sd4h.ca/en/overview; requests are reviewed under BQC19’s governance and require an approved data-use agreement.
