# Supplementary material for: Comparative plasma metabolomics of Delta and Omicron SARS-CoV-2 variants: insights into variant-specific pathogenesis and therapeutic implications
Source: Front Cell Infect Microbiol. 2025 Oct 16;15:1649724. doi: 10.3389/fcimb.2025.1649724 (PMC12576296; doi:10.3389/fcimb.2025.1649724)
Supplement: Supplementary file 1 [file DataSheet1.pdf]

Comparative Plasma Metabolomics of Delta and Omicron SARS-CoV-2 Variants: Insights into Variant-Specific Pathogenesis and Therapeutic Implications

SUPPLEMENTAL INFORMATION

Eric Pimentel,[a] Mohammad Mehdi Banoei,[a,b] Chel Hee Lee, [a,c] and Brent W. Winston [a,d] †

[a] Department of Critical Care, Cumming School of Medicine, University of Calgary, Calgary, AB, Canada.

[b] Department of Biomedical Engineering, Schulich School of Engineering, University of Calgary, Calgary.

[c] Department of Mathematics and Statistics, Faculty of Science, University of Calgary, Calgary, AB, Canada.

[d] Departments of Medicine and Biochemistry and Molecular Biology, Cumming School of Medicine, University of Calgary, Calgary, AB, Canada.

† Corresponding Author:

Brent W. Winston, MD, Departments of Critical Care Medicine, Medicine and Biochemistry and Molecular Biology, University of Calgary, Health Research Innovation Center (HRIC), Room 4C64, 3280 Hospital Drive N.W., Calgary, Alberta, Canada, T2N 4Z6.

Tel : (403) 220-4341

Fax : (403) 283-1267

Email: bwinston@ucalgary.ca

SUPPLEMENTAL METHODS' SECTION

Organic Quantification

For organic acid quantification, 50 µL plasma was thawed on ice, followed by the addition of 150 µL of ice-cold methanol and 10 µL of isotope-labeled standards. The mixtures were kept overnight at -20 °C to precipitate proteins, then centrifuged at 13,000×g for 20 minutes. A total of 50 µL of supernatant was transferred to each well of a 96-well plate, followed by adding a 3-nitrophenylhydrazine reagent for extraction and incubation for two hours. Butylated hydroxytoluene (2 mg/ml), stabilizer, and water were added to the extract before the LC-MS/MS injection (1).

Amino acid and lipid Quantifications

For amino acid and lipid quantifications, samples were vortexed and centrifuged. Subsequently, 10 µL of each sample was added to a 96-well plate and dried under a stream of nitrogen. A phenyl-isothiocyanate reagent was added to the samples on the plate to derivatize the sample molecules. The samples were incubated and then dried using an evaporator. Following this, 300 µL of extraction solvent was added to the analytes. Extracts were centrifuged to the lower part of the 96-well plate, and a dilution step was performed using 0.2% formic acid in water and 0.2% formic acid in acetonitrile (2). Samples were then loaded for LC-MS/MS analysis. The LC-MS/MS analysis parameters were the following: Mobile phase A was 0.2% (v/v) formic acid in water, and mobile phase B was 0.2% (v/v) formic acid in acetonitrile. The gradient parameters were t=0 min, 0% B; t=0.5 min, 0% B; $t=5.5$ min, 95% B; $t=6.5$ min, 95% B; $t=7.0$ min, 0% B; and t=9.5 min, 0% B. The chromatography column was set at 50°C. Ten microliters of sample were injected into the column with a flow rate of 300µl/min.

For chromatography of organic acids, mobile phase A was 0.01% (v/v) formic acid in water, and mobile phase B was 0.01% (v/v) formic acid in methanol. The gradient parameters were $t=0$ min, 30% B; t=2.0 min, 50% B; t=12.5 min, 95% B; t=12.5 min, 100% B; t=13.5min, 100% B; and t=13.6 min, and finally 30% B for 4.4 min. The chromatography column was set as 40°C. Ten microliters of samples were injected into the column with a flow rate of 300μl/min (3).

Data processing

Metabolites with concentrations below the limit of detection (LOD) were replaced by their respective LOD values. Metabolites were excluded if they contained insufficient reliable data according to two criteria: (1) metabolites were detected in fewer than 10% of samples (i.e., >90% missing or imputed data), or (2) metabolites where more than 10% of the samples required LOD imputation on any of the sampling days (day 1, 2 or 7). Additionally, glucose and citric acid were removed due to elevated levels from ACD collection tubes. After applying these exclusion criteria, 102 metabolites were included in the analysis. See Supplemental Table 1 (Table S1) for specific details on included/excluded metabolites. For subsequent analyses, a base-10 logarithmic transformation and unit variance scaling were applied to the remaining metabolites in order to normalize data distribution and ensure comparability among metabolites with different concentration ranges.

Two-way ANOVA

Separate two-way ANOVAs for each sampling day (days 1, 2, 7) to examine interaction effects between three factor pairs: (i) clinical severity (m+M vs. S+C) × variant (Delta vs. Omicron), (ii) corticosteroid treatment (no/yes) × variant, and (iii) vaccination completeness (<2 vs. ≥2 doses) × variant was performed. Assumptions were addressed as follows: normality of the transformed concentrations was checked with a Shapiro–Wilk test, and

homoscedasticity was addressed by unit-variance scaling, which standardizes variability across metabolites so that each contributes equally to the analysis. For each metabolite and day, Pr(>F)3 for the interaction effect was reported. *Pr(>F)3* is the ANOVA p-value associated with the F-statistic that tests whether the between-group variance explained by the interaction is greater than expected under the null hypothesis of no interaction (i.e., no difference among the factor-level means beyond random variation).

SUPPLEMENTARY LIMITATIONS

Our observational, retrospective design is subject to selection bias and confounding from unmeasured or incompletely measured factors (e.g., diet, fasting status, time of day, and other pre-analytical variables that are known to shift plasma metabolite levels). Pre-collection and handling effects, including fasting duration, circadian timing, and short processing delays, can materially alter metabolomic readouts, and we could not standardize all these factors across sites. (4)

Corticosteroid exposure and vaccine status may also modulate systemic metabolism independent of infection; corticosteroids have well-documented, broad metabolomic effects in humans, and product/timing of vaccination were inconsistently recorded, limiting adjustment beyond our completeness categories (5).

Comorbidity-related metabolic signatures and implications for adjustment. Several common comorbidities carried by hospitalized COVID-19 patients have distinct plasma metabolomic fingerprints that can mimic or obscure variant-associated signals. For example, obesity, insulin resistance, and type 2 diabetes are repeatedly associated with elevations in branched-chain amino acids and lipids (6). Chronic kidney disease (CKD) is characterized by retention of uremic solutes and broad shifts in amino-acid and energy

pathways, which alter the circulating metabolome even in the absence of acute infection (7). Chronic lung disease features changes across amino-acid and lipid metabolism, including altered acylcarnitines and amino acids that can overlap with systemic inflammatory states (8). Taken together, these disease-specific signatures increase the risk that observed differences between variants partially reflect baseline comorbidity biology rather than variant effects alone.

Finally, disease time course is another constraint: viral load peaks near symptom onset and typically declines by the time of hospitalization; we did not have patient-level viral-load data and therefore could not adjust for it, so some metabolic differences could reflect viral-load stage rather than variant per se (9, 10).

SUPPLEMENTAL TABLES AND FIGURES

Table S1. Contains list of metabolites developed by The Metabolomics Innovations Center of the University of Alberta (143 metabolites), TMIC Prime assay. Metabolites excluded due to high proportion of LOD and unique values exceeding 10% are also shown.

	Name	HMDB ID	Metabolite excluded	LOD values >10%	# of unique values >10%
1	Creatinine	HMDB0000562	No		
2	Glycine	HMDB0000123	No		
3	Alanine	HMDB0000161	No		
4	Serine	HMDB0000187	No		
5	Histamine	HMDB0000870	Yes		Yes
6	Proline	HMDB0000162	No		
7	Valine	HMDB0000883	No		
8	Threonine	HMDB0000167	No		
9	Phenylethylamine	HMDB0012275	Yes		Yes
10	Taurine	HMDB0000251	No		
11	Putrescine	HMDB0001414	Yes	Yes	
12	cis-Hydroxyproline	HMDB0240251	Yes		Yes
13	trans-Hydroxyproline	HMDB0000725	No		
14	Leucine	HMDB0000687	No		
15	Isoleucine	HMDB0000172	No		
16	Asparagine	HMDB0000168	No		
17	Aspartic acid	HMDB0000191	No		
18	Glutamine	HMDB0000641	No		
19	Glutamic acid	HMDB0000148	No		
20	Methionine	HMDB0000696	No		
21	Dopamine	HMDB0000073	Yes		Yes
22	Histidine	HMDB0000177	No		
23	alpha-aminoadipic acid	HMDB0000510	No		
24	Phenylalanine	HMDB0000159	No		
25	Methionine-Sulfoxide	HMDB0002005	No		
26	Arginine	HMDB0000517	No		

27	Acetyl-ornithine	HMDB0003357	No		
28	Citrulline	HMDB0000904	No		
29	Serotonin	HMDB0000259	Yes	Yes	
30	Tyrosine	HMDB0000158	No		
31	DOPA	HMDB0000181	Yes		Yes
32	Asymmetric dimethylarginine	HMDB0001539	No		
33	Total dimethylarginine	HMDB0251395	No		
34	Tryptophan	HMDB0000929	No		
35	Kynurenine	HMDB0000684	No		
36	Carnosine	HMDB0000033	Yes		Yes
37	Nitro-Tyrosine	HMDB0001904	Yes		Yes
38	Ornithine	HMDB0000214	No		
39	Lysine	HMDB0000182	No		
40	Spermidine	HMDB0001257	No		
41	Spermine	HMDB0001256	No		
42	Sarcosine	HMDB0000271	Yes	Yes	
43	Diacetylspermine	HMDB0002172	Yes	Yes	Yes
44	Tyramine	HMDB0000306	Yes		Yes
45	Creatine	HMDB0000064	No		
46	Phosphocreatine	HMDB0001511	Yes		Yes
47	Betaine	HMDB0000043	No		
48	Choline	HMDB0000097	No		
49	Trimethylamine N-oxide	HMDB0000925	Yes	Yes	
50	Methylhistidine (	HMDB0000001	No		
51	Lactic acid	HMDB0000190	No		
52	Beta-Hydroxybutyric acid	HMDB0000011	No		
53	Alpha-Ketoglutaric acid	HMDB0000208	No		
54	Citric acid	HMDB0000094	Present in ACD tubes		
55	Butyric acid	HMDB0000039	No		
56	Propionic acid	HMDB0000237	No		
57	HPHPA	HMDB0002643	Yes		Yes

58	para-Hydroxyhippuric acid	HMDB0013678	No		
59	Succinic acid	HMDB0000254	No		
60	Fumaric acid	HMDB0000134	No		
61	Pyruvic acid	HMDB0000243	No		
62	Isobutyric acid	HMDB0001873	No		
63	Hippuric acid	HMDB0000714	No		
64	Methylmalonic acid	HMDB0000202	No		
65	Homovanillic acid	HMDB0000118	No		
66	Homocysteine	HMDB0000742	No		
67	Indole acetic acid	HMDB0000197	No		
68	Uric acid	HMDB0000289	No		
69	Glucose	HMDB0000122	Present in ACD tubes		
70	LysoPC a C14:0	HMDB0010379	No		
71	LysoPC a C16:1	HMDB0010383	No		
72	LysoPC a C16:0	HMDB0010382	No		
73	LysoPC a C17:0	HMDB0012108	No		
74	LysoPC a C18:2	HMDB0010386	No		
75	LysoPC a C18:1	HMDB0010385	No		
76	LysoPC a C18:0	HMDB0010384	No		
77	LysoPC a C20:4	HMDB0010396	No		
78	LysoPC a C20:3	HMDB0010394	No		
79	LysoPC a C24:0	HMDB0010405	Yes	Yes	
80	LysoPC a C26:1	HMDB0029220	No		
81	LysoPC a C26:0	HMDB0029205	No		
82	LysoPC a C28:1	HMDB0029221	No		
83	LysoPC a C28:0	HMDB0029206	No		
84	SM(OH) C14:1	HMDB0013462	No		
85	SM C16:1	HMDB0240613	No		
86	SM C16:0	HMDB0010169	No		
87	SM(OH) C16:1	HMDB0013463	No		
88	SM C18:1	HMDB0012100	No		

89	PC aa C32:2	HMDB0007874	No		
90	SM C18:0	HMDB0001348	No		
91	SM C20:2	HMDB0013465	No		
92	PC ae C36:0	HMDB0013406	No		
93	PC aa C36:6	HMDB0008690	No		
94	PC aa C36:0	HMDB0007886	No		
95	SM(OH) C22:2	HMDB0013467	No		
96	SM(OH) C22:1	HMDB0013466	No		
97	PC aa C38:6	HMDB0007991	No		
98	PC aa C38:0	HMDB0007893	No		
99	PC ae C40:6	HMDB0013422	No		
100	SM(OH) C24:1	HMDB0013469	No		
101	PC aa C40:6	HMDB0008057	No		
102	PC aa C40:2	HMDB0008688	No		
103	PC aa C40:1	HMDB0007993	No		
104	C0	HMDB0000062	No		
105	C2	HMDB0000201	No		
106	C3:1	HMDB0013124	Yes	Yes	
107	C3	HMDB0000824	No		
108	C4:1	HMDB0013126	Yes	Yes	
109	C4	HMDB0002013	No		
110	C3OH	HMDB0013125	Yes	Yes	
111	C5:1	HMDB0002366	No		
112	C5	HMDB0013128	No		
113	C4OH	HMDB0013127	No		
114	C6:1	HMDB0013161	Yes	Yes	
115	C6	HMDB0000756	Yes	Yes	
116	C5OH	HMDB0013132	Yes	Yes	
117	C5:1DC	HMDB0013129	Yes	Yes	
118	C5DC	HMDB0013130	No		
119	C8	HMDB0000791	No		
120	C5MDC	HMDB0000552	Yes	Yes	

121	C9	HMDB0013288	No		
122	C7DC	HMDB0013328	No		
123	C10:2	HMDB0241102	Yes	Yes	
124	C10:1	HMDB0013205	No		
125	C10	HMDB0000651	No		
126	C12:1	HMDB0013326	No		
127	C12	HMDB0002250	Yes	Yes	
128	C14:2	HMDB0013331	Yes	Yes	
129	C14:1	HMDB0002014	Yes	Yes	
130	C14	HMDB0005066	Yes	Yes	
131	C12DC	HMDB0013327	No		
132	C14:2OH	HMDB0013332	Yes		Yes
133	C14:1OH	HMDB0013330	No		
134	C16:2	HMDB0013334	Yes	Yes	Yes
135	C16:1	HMDB0013207	Yes	Yes	
136	C16	HMDB0000222	Yes	Yes	
137	C16:2OH	HMDB0013335	Yes	Yes	
138	C16:1OH	HMDB0013333	Yes	Yes	Yes
139	C16OH	HMDB0013336	Yes		Yes
140	C18:2	HMDB0006469	Yes	Yes	
141	C18:1	HMDB0094687	No		
142	C18	HMDB0000848	Yes	Yes	
143	C18:1OH	HMDB0013340	Yes		Yes

Table S1.1 Variant-associated fold changes (Delta vs. Omicron) by sampling day. Metabolites showing the largest and/or statistically supported differences between variants are listed for hospital Day 1, Day 2, and Day 7. Columns indicate the direction of change and the fold change (FC). "Increased" denotes higher abundance in Delta relative to Omicron (FC > 1), whereas "Decreased" denotes higher abundance in Omicron (FC < 1).

	Increased	FC	Decreased	FC
DAY 1	LysoPC a C26:0	1.472	Aspartic acid	0.764
	LysoPC a C28:0	1.325	Spermidine	0.788
	PC aa C38:6	1.232	beta-Hydroxybutiric acid	0.789
	LysoPC a C18:2	1.209	Taurine	0.799
	Threonine	1.204	Spermine	0.809
	LysoPC a C20:3	1.193	Acetyl-ornithine	0.883
	LysoPC a C20:4	1.18		
	Asparagine	1.18		
	Glutamine	1.149		
DAY 2	PC ae C:36:0	1.236	Acetyl-ornithine	0.642
	Threonine	1.236	Aspartic acid	0.642
	C3	1.16	Betaine	0.711
	Pyruvic acid	1.16	PC aa C36:0	0.778
	Lyso PC a C16:0	1.137	Leucine	0.827
	Mehionine-sulfoxide	1.139	Citrulline	0.832
	Total dimethylarginine	1.136	C18:1	0.865
			Homocysteine	0.906
DAY 7	Indole acetic acid	1.382	Isobutyric acid	0.768
	C4	1.374	Spermine	0.801
	Kynurenine	1.272	Citrulline	0.905
	C2	1.267	Propionic acid	0.929
	C4OH	1.251	Spermidine	0.929
	Isoleucine	1.233		
	Leucine	1.211		
	PC ae C40:6	1.157		
	C10	1.226		
	Tyrosine	1.148		
	Asymmetric dimethylarginine	1.103		
	C5	1.171		

Figure S1a. Summary of median metabolomic concentration changes (↑/↓) differentiating Delta and Omicron variants on days 1, 2, and 7 of hospitalization.

Delta vs Omicron																						
Day/ Metabolite	Acetyl-ornithine	Asparagine	Aspartic acid	Asymmetric dimethylarginine	C10	C18:1	C3	C4	C4OH	C5	Glutamine	Homocysteine	Isoleucine	Leucine	LysoPC a C18:2	LysoPC a C20:3	LysoPC a C20:4	PC aa C38:6	PC ae C40:6	Taurine	Threonine	Tyrosine
1	↓	↑	↓								↑				↑	↑	↑	↑		↓	↑	
2	↓					↓	↑					↓									↑	
7				↑	↑			↑	↑	↑			↑	↑					↑			↑

↑ ↓ Delta compared to Omicron

Figure S1b. Summary of median metabolomic concentration changes (↑/↓) differentiating mild plus moderate and severe plus critical groups on days 1, 2, and 7 of hospitalization.

Severity NIH classification (mild+Moderate vs Severe + Critical)																																
Day/ Metabolite	Acetyl-ornithine	Alanine	alpha-Aminoadipic acid	alpha-Ketoglutaric acid	Asparagine	Aspartic acid	C0	C3	C4	C5	Creatine	Fumaric acid	Glutamine	Homocysteine	Homovanillic acid	Kynurenine	Lactic acid	Leucine	Lysine	LysoPC a C20:4	Methionine	Methionine-sulfoxide	Ornithine	PC aa C32:2	Phenylalanine	Pyruvic acid	Succinic acid	Threonine	trans-hydroxyproline	Tryptophan	Tyrosine	Valine
1		↑	↑		↑	↑	↑	↑		↑	↑		↑	↓					↑	↑						↑		↑				↑
2			↑		↑	↓	↑	↑	↑	↑	↑				↑	↑			↑		↑	↑	↑		↑				↓		↑	
7	↓	↑		↑			↑	↑	↑	↑	↑	↑				↑	↑	↑	↑		↑			↑	↑	↑	↑		↓	↑	↑	↑

↑ ↓ S+C compared to m+M

Figure S1c. Summary of median metabolomic concentration changes (↑/↓) differentiating corticosteroid and non-corticosteroid use groups on days 1, 2, and 7 of hospitalization.

Corticosteroid vs Non-corticosteroid use																										
Day/ Metabolite	Acetyl-ornithine	Alanine	alpha-Aminoadipic acid	alpha-Ketoglutaric acid	Asparagine	Asymmetric dimethylarginine	beta-Hydroxybutyric acid	C3	C4	C5	Fumaric acid	Glutamine	Indole acetic acid	Leucine	Lysine	LysoPC a C18:0	Methionine	PC aa C32:2	Phenylalanine	SM(OH) C14:1	SM(OH) C22:1	Threonine	Total dimethylarginine	Tryptophan	Tyrosine	Valine
1	↓	↑				↓						↑					↑				↑	↑	↓			
2		↑	↑		↑			↑	↑	↑					↑		↑									
7	↓	↑		↑			↑				↑		↓	↑	↑	↓	↑	↑	↑	↑				↑	↑	↑

↑ ↓ corticosteroid compared to non-corticosteroid use

Figure S1d. Summary of median metabolomic concentration changes (↑/↓) differentiating complete and incomplete vaccination groups on days 1, 2, and 7 of hospitalization.

Complete vs incomplete vaccination																								
Day/ Metabolite	Acetyl-ornithine	alpha-Aminoadipic acid	alpha-Ketoglutaric acid	Asparagine	C10:1	C3	C4	C4OH	C9	Citrulline	Glycine	Isobutyric acid	Lysine	LysoPC a C26:1	Methionine	PC aa C36:6	Proline	Propionic acid	Pyruvic acid	SM C20:2	Taurine	Threonine	trans-Hydroxyproline	Uric acid
1		↓	↓	↓		↓						↑	↓		↓		↓	↑	↓		↑	↓		
2						↓		↑		↑								↑			↑		↑	↑
7	↑				↑	↓	↓			↑	↑			↓		↓				↓			↑	

↑ ↓ Complete compared to incomplete vaccination

Table S2. Pathway analysis of significant metabolites associated with variant differentiation, COVID-19 severity, corticosteroid treatment, and vaccination status.

**Pathway analysis associated with variant differentiation**	**Raw p**	**log 10(p)**	**Holm adjust**	**FDR**	**Impact**
Valine, leucine and isoleucine biosynthesis	0.000	4.993	0.001	0.001	0.000
Arginine biosynthesis	0.000	4.189	0.005	0.003	0.000
Alanine, aspartate and glutamate metabolism	0.001	3.255	0.043	0.015	0.337
Valine, leucine and isoleucine degradation	0.025	1.604	1.000	0.403	0.000
Phenylalanine, tyrosine and tryptophan biosynthesis	0.025	1.599	1.000	0.403	0.500
Taurine and hypotaurine metabolism	0.050	1.303	1.000	0.498	0.429
**Pathway analysis related to COVID-19 severity**					
Alanine, aspartate and glutamate metabolism	0.000	8.523	0.000	0.000	0.388
Arginine biosynthesis	0.000	7.633	0.000	0.000	0.061
Valine, leucine and isoleucine biosynthesis	0.000	3.726	0.015	0.004	0.000
Citrate cycle (TCA cycle)	0.000	3.695	0.016	0.004	0.167
Phenylalanine, tyrosine and tryptophan biosynthesis	0.001	2.847	0.108	0.023	1.000
**Pathway analysis associated with corticosteroid treatment**					
Alanine, aspartate and glutamate metabolism	0.000	5.185	0.001	0.000	0.164
Arginine biosynthesis	0.000	5.059	0.001	0.000	0.000
Valine, leucine and isoleucine biosynthesis	0.000	4.247	0.004	0.002	0.000
Phenylalanine metabolism	0.003	2.529	0.225	0.047	0.357
Phenylalanine, tyrosine and tryptophan biosynthesis	0.001	3.187	0.050	0.013	1.000
**Pathway analysis associated with vaccination status**					
Arginine biosynthesis	0.000	3.621	0.019	0.019	0.228
Alanine, aspartate and glutamate metabolism	0.001	2.701	0.156	0.052	0.048
Lipoic acid metabolism	0.001	2.701	0.156	0.052	0.001
Glycine, serine and threonine metabolism	0.003	2.492	0.247	0.064	0.259
Arginine and proline metabolism	0.004	2.383	0.314	0.066	0.038

**Figure S2a. Pathway analysis of metabolites associated with variant differences**	**Figure S2b. Pathway analysis of metabolites associated with COVID-19 severity**
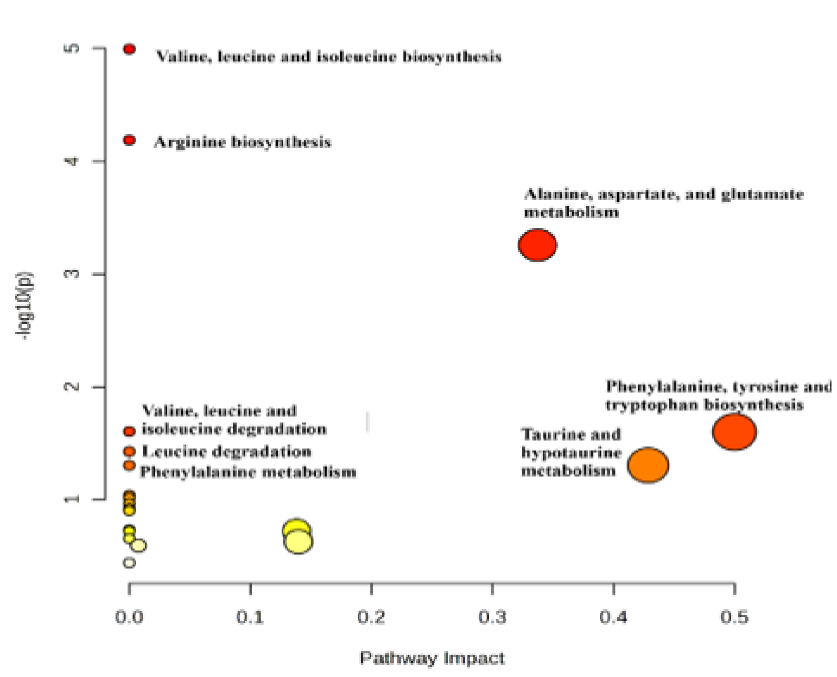	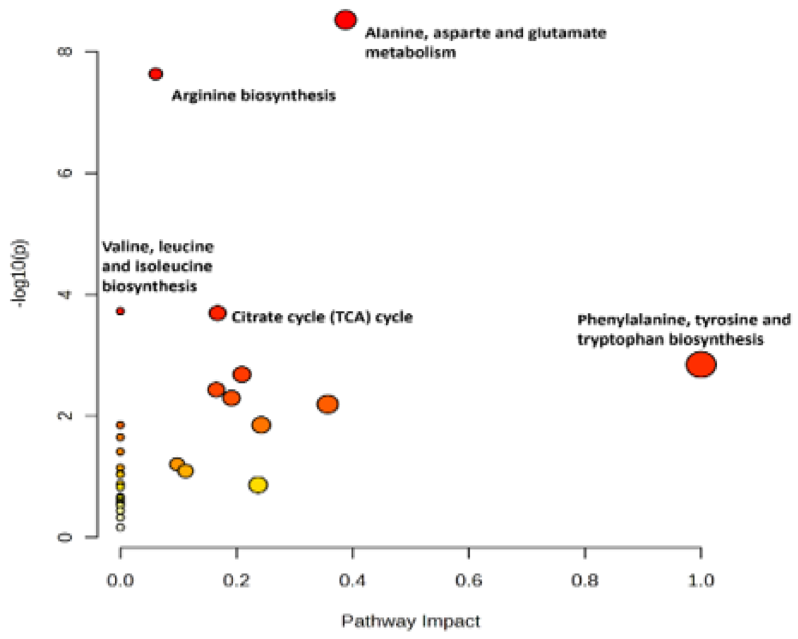

Figure S2c. Pathway analysis of metabolites associated with corticosteroid treatment.

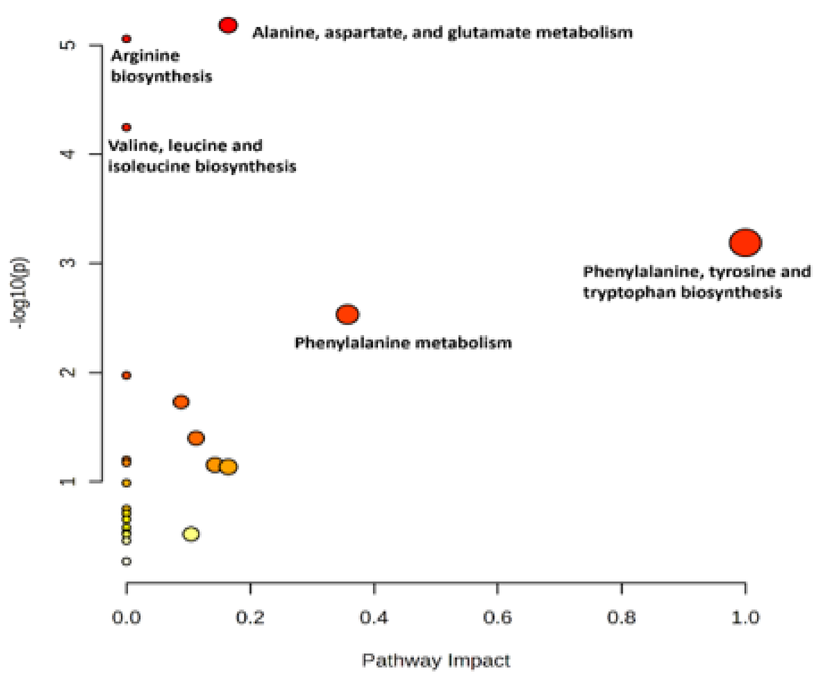

Figure S2d. Pathway analysis of metabolites associated with vaccination.

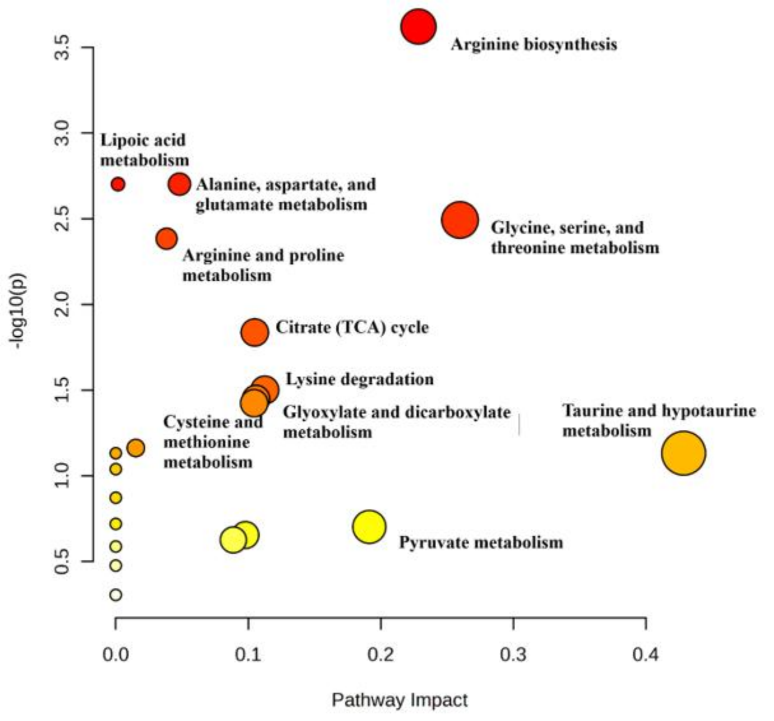

Table S3. Two-way ANOVA results for the effects of severity and SARS-CoV-2 variant on plasma metabolites across days 1, 2, and 7. Pr(>F) 3 represents the p-value for the interaction effect between severity and SARS-CoV-2 variant. Metabolites with significant interaction effects (p<0.05) on at least one day (1,2, or 7) are highlighted with an asterisk (*).

**Metabolite**	**Day 1 Pr(>F)3**	**Day 2 Pr(>F)3**	**Day 7 Pr(>F)3**
Acetyl-ornithine	0.478	0.833	0.333
Alanine	0.054	0.600	0.480
alpha-Aminoadipic acid	0.335	0.841	0.867
alpha-Ketoglutaric acid	0.933	0.660	0.497
Arginine	0.561	0.394	0.945
Asparagine	0.815	0.411	0.086
Aspartic acid	0.613	0.074	0.701
Asymmetric dimethylarginine *	0.043	0.081	0.210
beta-Hydroxybutyric acid	0.858	0.297	0.105
Betaine *	0.010	0.100	0.150
Butyric acid	0.610	0.541	0.274
C0	0.951	0.814	0.659
C10	0.223	0.335	0.285
C10:1	0.944	0.260	0.447
C12:1	0.502	0.948	0.227
C18:1	0.483	0.609	0.626
C2	0.679	0.406	0.348
C3	0.557	0.721	0.491
C4	0.485	0.783	0.328
C4OH	0.757	0.782	0.298
C5	0.461	0.232	0.381
C5DC	0.402	0.472	0.708

C7DC	0.735	0.692	0.535
C8	0.554	0.535	0.852
C9	0.349	0.486	0.832
Choline	0.208	0.086	0.425
Citrulline	0.143	0.152	0.215
Creatine *	0.006	0.323	0.350
Creatinine *	0.445	0.006	0.721
Fumaric acid *	0.302	0.215	0.025
Glutamic acid	0.393	0.355	0.112
Glutamine	0.256	0.347	0.211
Glycine	0.814	0.777	0.321
Hippuric acid	0.254	0.078	0.326
Histidine	0.588	0.282	0.219
Homocysteine	0.192	0.388	0.493
Homovanillic acid	0.829	0.137	0.181
Indole acetic acid	0.694	0.246	0.327
Isobutyric acid	0.635	0.250	0.761
Isoleucine *	0.013	0.030	0.019
Kynurenine	0.413	0.104	0.588
Lactic acid	0.220	0.422	0.212
Leucine *	0.008	0.027	0.112
Lysine *	0.028	0.703	0.027
LysoPC a C14:0	0.301	0.922	0.069
LysoPC a C16:0	0.460	0.981	0.224
LysoPC a C16:1	0.827	0.561	0.697
LysoPC a C17:0	0.574	0.674	0.662
LysoPC a C18:0 *	0.344	0.770	0.043
LysoPC a C18:1	0.417	0.281	0.646

LysoPC a C18:2	0.406	0.778	0.284
LysoPC a C20:3	0.468	0.986	0.725
LysoPC a C20:4	0.464	0.678	0.580
LysoPC a C26:0	0.591	0.827	0.982
LysoPC a C26:1	0.119	0.899	0.068
LysoPC a C28:0	0.366	0.378	0.477
LysoPC a C28:1	0.243	0.611	0.395
Methionine	0.786	0.515	0.403
Methionine-sulfoxide	0.451	0.567	0.157
Methylhistidine *	0.558	0.146	0.027
Methylmalonic acid	0.208	0.194	0.177
Ornithine	0.254	0.886	0.128
p-Hydroxyhippuric acid	0.108	0.417	0.259
PC aa C32:2	0.967	0.504	0.581
PC aa C36:0	0.847	0.312	0.519
PC aa C36:6	0.343	0.478	0.296
PC aa C38:0 *	0.414	0.008	0.041
PC aa C38:6	0.808	0.293	0.617
PC aa C40:1	0.185	0.069	0.168
PC aa C40:2	0.419	0.201	0.441
PC aa C40:6	0.827	0.667	0.802
PC ae C36:0	0.134	0.580	0.658
PC ae C40:6	0.298	0.080	0.460
Phenylalanine	0.990	0.613	0.071
Proline	0.147	0.309	0.469
Propionic acid	0.690	0.893	0.623
Pyruvic acid	0.466	0.685	0.371
Serine	0.786	0.655	0.766

SM C16:0	0.666	0.517	0.748
SM C16:1	0.348	0.083	0.890
SM C18:0	0.810	0.286	0.863
SM C18:1	0.323	0.183	0.832
SM C20:2	0.918	0.541	0.496
SM(OH) C14:1	0.907	0.201	0.306
SM(OH) C16:1	0.690	0.463	0.492
SM(OH) C22:1	0.843	0.100	0.982
SM(OH) C22:2	0.241	0.291	0.287
SM(OH) C24:1	0.111	0.107	0.592
Spermidine *	0.946	0.032	0.557
Spermine *	0.796	0.049	0.781
Succinic acid	0.936	0.250	0.578
Taurine	0.542	0.485	0.256
Threonine	0.546	0.733	0.135
Total dimethylarginine *	0.056	0.009	0.446
trans-Hydroxyproline	0.747	0.328	0.340
Tryptophan	0.271	0.391	0.217
Tyrosine	0.391	0.357	0.141
Uric acid	0.952	0.312	0.331
Valine *	0.002	0.059	0.129

Figure S3. Heatmap of significant interaction effects between severity and *SARS-CoV-2* variant on plasma metabolite concentrations. Mean metabolic concentrations across days 1,2, and 7, comparing mild plus moderate (m+M) versus severe plus critical (S+C) disease severity groups and Delta (D) versus Omicron (O) variants. The color scale indicates the relative ranking of mean metabolite levels (highest, second highest, second lowest, and lowest).

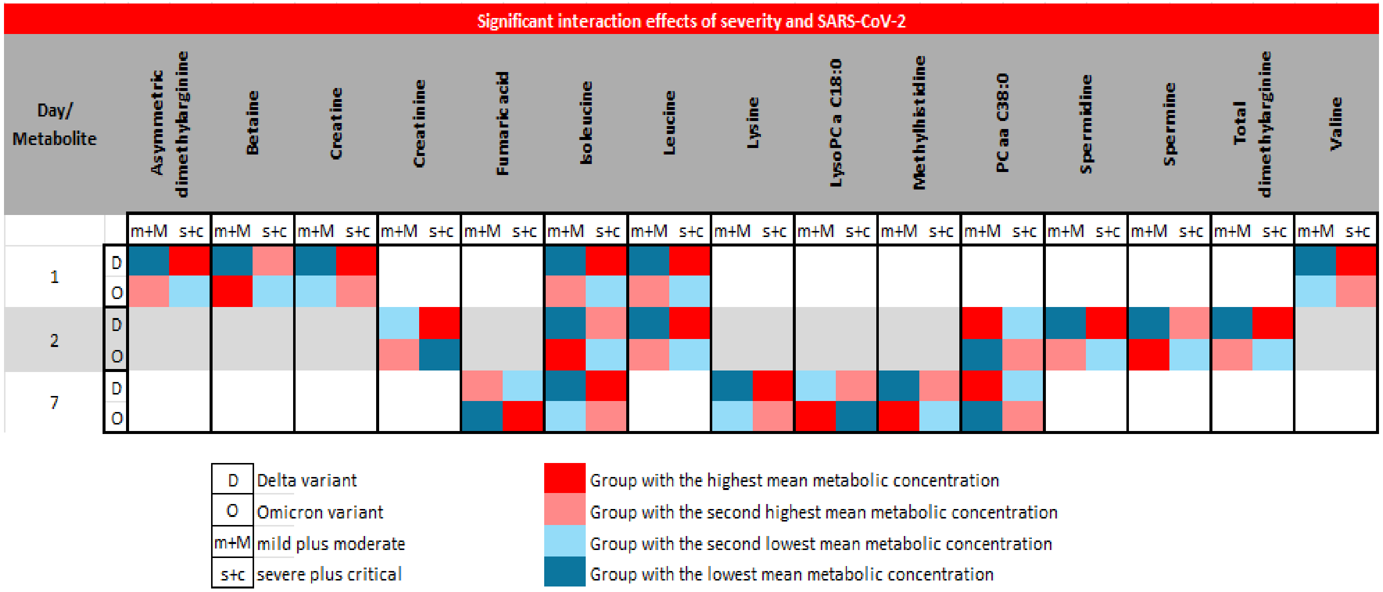

Table S4. Two-way ANOVA results for the effects of treatment and SARS-CoV-2 variant on plasma metabolites across days 1, 2, and 7. Pr(>F) 3 represents the p-value for the interaction effect between treatment and SARS-CoV-2 variant. Metabolites with significant interaction effects ($p<0.05$) on at least one day (1,2, or 7) are highlighted with an asterisk (*).

**Metabolite**	**Day 1 Pr(>F)3**	**Day 2 Pr(>F)3**	**Day 7 Pr(>F)3**
Acetyl-ornithine	0.175	0.335	0.453
Alanine	0.418	0.701	0.625
alpha-Aminoadipic acid	0.821	0.159	0.679
alpha-Ketoglutaric acid	0.579	0.424	0.121
Arginine	0.549	0.471	0.399
Asparagine	0.967	0.709	0.678
Aspartic acid	0.261	0.063	0.903
Asymmetric dimethylarginine	0.419	0.277	0.789
beta-Hydroxybutyric acid	0.616	0.505	0.359
Betaine	0.980	0.854	0.768
Butyric acid	0.977	0.521	0.976
C0 *	0.289	0.096	0.033
C10	0.551	0.607	0.260
C10:1	0.988	0.690	0.443
C12:1	0.671	0.843	0.945
C18:1	0.480	0.400	0.616
C2	0.796	0.387	0.954
C3	0.820	0.220	0.877
C4 *	0.106	0.029	0.327
C4OH	0.535	0.647	0.699
C5	0.949	0.144	0.218

C5DC	0.891	0.549	0.638
C7DC	0.693	0.718	0.567
C8	0.865	0.582	0.152
C9	0.556	0.708	0.977
Choline	0.760	0.733	0.100
Citrulline	0.417	0.455	0.427
Creatine	0.161	0.893	0.845
Creatinine	0.661	0.944	0.131
Fumaric acid	0.491	0.283	0.468
Glutamic acid	0.457	0.735	0.104
Glutamine	0.753	0.738	0.830
Glycine *	0.496	0.049	0.748
Hippuric acid	0.317	0.434	0.423
Histidine	0.809	0.415	0.443
Homocysteine *	0.557	0.502	0.021
Homovanillic acid	0.706	0.076	0.284
Indole acetic acid *	0.428	0.322	0.010
Isobutyric acid	0.780	0.217	0.696
Isoleucine	0.848	0.430	0.713
Kynurenine *	0.161	0.600	0.001
Lactic acid	0.051	0.150	0.670
Leucine	0.105	0.912	0.493
Lysine	0.733	0.923	0.674
LysoPC a C14:0 *	0.660	0.790	0.020
LysoPC a C16:0	0.558	0.500	0.689
LysoPC a C16:1	0.284	0.279	0.285
LysoPC a C17:0	0.464	0.317	0.983
LysoPC a C18:0	0.397	0.292	0.373

LysoPC a C18:1 *	0.041	0.335	0.911
LysoPC a C18:2	0.416	0.899	0.190
LysoPC a C20:3	0.957	0.448	0.590
LysoPC a C20:4	0.235	0.433	0.514
LysoPC a C26:0	0.543	0.590	0.285
LysoPC a C26:1	0.643	0.194	0.836
LysoPC a C28:0	0.445	0.424	0.317
LysoPC a C28:1	0.569	0.847	0.579
Methionine	0.519	0.506	0.962
Methionine-sulfoxide	0.456	0.083	0.727
Methylhistidine	0.421	0.680	0.461
Methylmalonic acid	0.510	0.460	0.941
Ornithine	0.468	0.960	0.934
p-Hydroxyhippuric acid *	0.252	0.629	0.024
PC aa C32:2	0.503	0.644	0.792
PC aa C36:0 *	0.504	0.853	0.023
PC aa C36:6	0.926	0.928	0.129
PC aa C38:0	0.517	0.739	0.684
PC aa C38:6	0.433	0.856	0.505
PC aa C40:1 *	0.176	0.656	0.026
PC aa C40:2	0.322	0.230	0.771
PC aa C40:6	0.243	0.666	0.332
PC ae C36:0	0.426	0.876	0.085
PC ae C40:6	0.110	0.385	0.158
Phenylalanine *	0.349	0.503	0.026
Proline	0.614	0.118	0.922
Propionic acid	0.871	0.431	0.800
Pyruvic acid	0.533	0.699	0.913

Serine	0.593	0.082	0.934
SM C16:0	0.799	0.930	0.520
SM C16:1	0.641	0.456	0.331
SM C18:0	0.579	0.662	0.984
SM C18:1	0.765	0.531	0.763
SM C20:2	0.885	0.607	0.959
SM(OH) C14:1	0.234	0.376	0.823
SM(OH) C16:1	0.547	0.415	0.459
SM(OH) C22:1	0.875	0.433	0.850
SM(OH) C22:2	0.523	0.859	0.556
SM(OH) C24:1 *	0.049	0.395	0.449
Spermidine	0.265	0.144	0.901
Spermine	0.068	0.112	0.512
Succinic acid	0.828	0.993	0.636
Taurine *	0.790	0.033	0.913
Threonine	0.295	0.357	0.662
Total dimethylarginine	0.877	0.737	0.159
trans-Hydroxyproline	0.552	0.295	0.677
Tryptophan	0.331	0.688	0.789
Tyrosine	0.690	0.404	0.076
Uric acid	0.981	0.410	0.484
Valine	0.278	0.725	0.424

Figure S4. Heatmap showing significant interaction effects of corticosteroid use and *SARS-CoV-2* variant on plasma metabolite concentrations across days 1, 2, and 7. The color scale indicates the relative ranking of mean metabolic concentrations (highest, second highest, second lowest, lowest) for each combination of variant (Delta vs. Omicron) and treatment status (non-corticosteroid use vs. corticosteroid use).

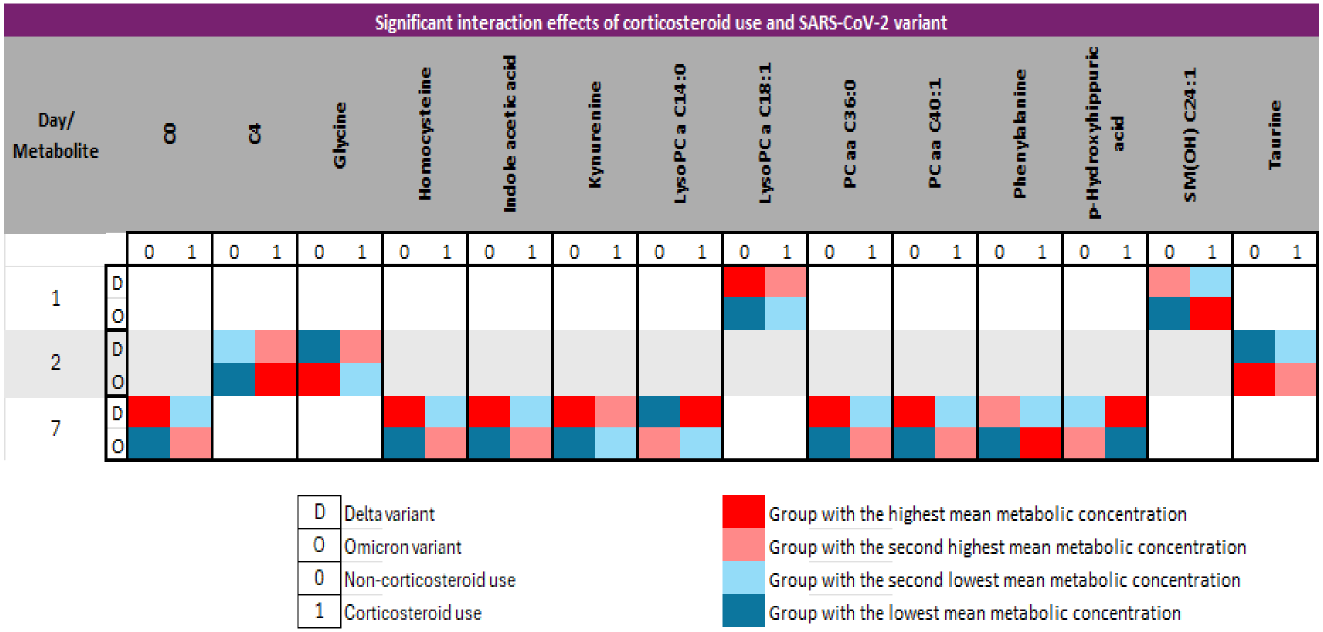

Table S5. Two-way ANOVA results for the effects of vaccination status and SARS-CoV-2 variant on plasma metabolites across days 1, 2, and 7. Pr(>F) 3 represents the p-value for the interaction effect between vaccination and SARS-CoV-2 variant. Metabolites with significant interaction effects ($p<0.05$) on at least one day (1,2, or 7) are highlighted with an asterisk (*).

**Metabolite**	**Day 1 Pr(>F)3**	**Day 2 Pr(>F)3**	**Day 7 Pr(>F)3**
Acetyl-ornithine	0.903	0.738	0.610
Alanine	0.087	0.460	0.271
alpha-Aminoadipic acid	0.471	0.650	0.251
alpha-Ketoglutaric acid	0.526	0.582	0.915
Arginine	0.598	0.700	0.600
Asparagine	0.388	0.523	0.918
Aspartic acid	0.227	0.694	0.846
Asymmetric dimethylarginine	0.558	0.613	0.138
beta-Hydroxybutyric acid	0.110	0.227	0.271
Betaine	0.747	0.690	0.473
Butyric acid	0.509	0.322	0.768
C0	0.821	0.698	0.502
C10	0.753	0.999	0.522
C10:1	0.932	0.462	0.339
C12:1	0.466	0.293	0.979
C18:1	0.693	0.898	0.132
C2	0.746	0.411	0.716
C3	0.568	0.176	0.475
C4	0.566	0.245	0.584
C4OH	0.825	0.179	0.070
C5	0.744	0.573	0.762
C5DC *	0.771	0.528	0.037

C7DC *	0.647	0.205	0.003
C8	0.295	0.724	0.633
C9	0.337	0.925	0.286
Choline	0.920	0.360	0.782
Citrulline	0.547	0.913	0.629
Creatine	0.573	0.844	0.680
Creatinine	0.432	0.235	0.346
Fumaric acid	0.806	0.976	0.736
Glutamic acid	0.110	0.159	0.872
Glutamine	0.113	0.597	0.725
Glycine	0.432	0.360	0.569
Hippuric acid	0.694	0.460	0.513
Histidine	0.665	0.769	0.861
Homocysteine	0.800	0.196	0.502
Homovanillic acid	0.534	0.491	0.769
Indole acetic acid	0.253	0.514	0.485
Isobutyric acid	0.365	0.934	0.189
Isoleucine	0.413	0.060	0.959
Kynurenine	0.088	0.230	0.737
Lactic acid	0.212	0.544	0.587
Leucine	0.204	0.137	0.840
Lysine	0.976	0.656	0.210
LysoPC a C14:0	0.850	0.270	0.310
LysoPC a C16:0	0.103	0.893	0.618
LysoPC a C16:1	0.494	0.313	0.109
LysoPC a C17:0	0.229	0.473	0.288
LysoPC a C18:0	0.663	0.588	0.894
LysoPC a C18:1	0.647	0.682	0.871

LysoPC a C18:2	0.160	0.339	0.785
LysoPC a C20:3	0.342	0.682	0.305
LysoPC a C20:4	0.517	0.738	0.733
LysoPC a C26:0	0.688	0.509	0.228
LysoPC a C26:1	0.757	0.940	0.680
LysoPC a C28:0	0.847	0.401	0.638
LysoPC a C28:1	0.315	0.196	0.205
Methionine	0.739	0.933	0.641
Methionine-sulfoxide	0.810	0.733	0.414
Methylhistidine	0.753	0.801	0.715
Methylmalonic acid	0.985	0.262	0.349
Ornithine	0.917	0.518	0.211
PC aa C32:2	0.859	0.861	0.993
PC aa C36:0	0.374	0.103	0.484
PC aa C36:6	0.981	0.535	0.835
PC aa C38:0	0.323	0.132	0.732
PC aa C38:6	0.702	0.254	0.584
PC aa C40:1	0.852	0.456	0.577
PC aa C40:2	0.390	0.291	0.399
PC aa C40:6	0.484	0.742	0.873
PC ae C36:0	0.860	0.856	0.938
PC ae C40:6	0.599	0.263	0.184
Phenylalanine	0.739	0.569	0.991
p-Hydroxyhippuric acid	0.602	0.749	0.255
Proline	0.655	0.908	0.202
Propionic acid	0.866	0.501	0.564
Pyruvic acid	0.873	0.385	0.851
Serine	0.747	0.299	0.914

SM C16:0	0.386	0.901	0.620
SM C16:1	0.281	0.649	0.921
SM C18:0	0.390	0.771	0.826
SM C18:1	0.534	0.637	0.601
SM C20:2	0.580	0.607	0.608
SM(OH) C14:1	0.639	0.697	0.340
SM(OH) C16:1	0.391	0.806	0.511
SM(OH) C22:1	0.806	0.468	0.730
SM(OH) C22:2	0.304	0.664	0.947
SM(OH) C24:1	0.068	0.454	0.590
Spermidine	0.474	0.135	0.663
Spermine	0.820	0.167	0.609
Succinic acid	0.264	0.245	0.728
Taurine *	0.040	0.871	0.793
Threonine	0.535	0.938	0.392
Total dimethylarginine	0.599	0.052	0.894
trans-Hydroxyproline	0.828	0.940	0.352
Tryptophan	0.773	0.318	0.181
Tyrosine	0.214	0.706	0.857
Uric acid	0.662	0.679	0.679
Valine	0.188	0.353	0.745

Figure S5. Heatmap showing significant interaction effects of vaccination status and *SARS-CoV-2* variant on plasma metabolite concentrations across days 1, 2, and 7. The color scale indicates the relative ranking of mean metabolic concentrations (highest, second highest, second lowest, lowest) for each combination of variant (Delta vs. Omicron) and vaccination status (incomplete vaccination vs. complete vaccination).

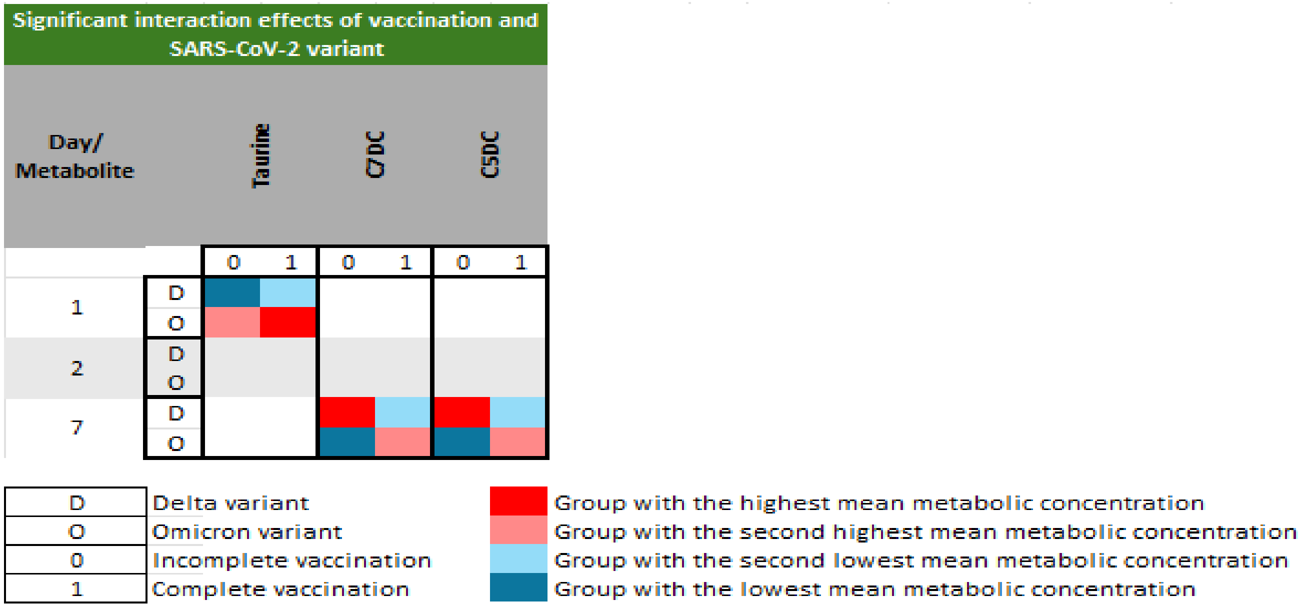

Table S6. In-hospital conditions by variant: counts, missingness, and unadjusted risk differences (Delta vs Omicron). "Delta: cases/53" and "Omicron: cases/56" show counts out of cohort sizes (53, 56). "Missing %" is the share without data for that condition in each variant. RD (pp) = $P(condition|Delta) - P(condition|Omicron)$ with 95% CI, calculated using observed non-missing denominators per condition. Analyses are exploratory and unadjusted. Abbrev: **RD** risk difference, **CI** confidence interval, **pp** percentage points.

Condition	Delta: cases/53	Delta: Missing % (of 53)	Omicron: cases/56	Omicron: Missing % (of 56)	Risk difference, pp (95% CI)
Hyperglycemia	15/53	1.9%	8/56	5.4%	12.7 (-9.4–33.0)
Liver dysfunction	8/53	0.0%	4/56	5.4%	7.5 (-10.0–24.1)
Viral pneumonia/pneumonitis	17/53	0.0%	13/56	5.4%	7.5 (-16.5–30.5)
Deep vein thrombosis	2/53	1.9%	0/56	7.1%	3.7 (-5.9–12.5)
Ventricular tachycardia or fibrillation (VT/VF)	1/53	0.0%	0/56	5.4%	1.9 (-6.4–9.9)
Seizure	1/53	1.9%	0/56	5.4%	1.9 (-6.4–9.8)
Cardiac arrest	2/53	1.9%	1/56	7.1%	1.8 (-9.1–12.2)
Acute kidney injury	8/53	1.9%	7/56	5.4%	1.6 (-17.1–20.1)
Anemia	15/53	1.9%	14/56	7.1%	0.9 (-22.6–24.1)
Bacterial pneumonia	12/53	1.9%	11/56	8.9%	0.7 (-21.4–22.4)
Pericarditis	0/53	1.9%	0/56	5.4%	0.0 (-6.8–6.6)
Myocarditis	0/53	1.9%	0/56	7.1%	0.0 (-6.9–6.6)
Rhabdomyolysis or myositis	0/53	1.9%	0/56	5.4%	0.0 (-6.8–6.6)
ST-elevation myocardial infarction (STEMI)	0/53	1.9%	0/56	5.4%	0.0 (-6.8–6.6)
TIA	0/53	1.9%	0/56	7.1%	0.0 (-6.9–6.6)
Gastrointestinal haemorrhage	0/53	1.9%	0/56	5.4%	0.0 (-6.8–6.6)
Endocarditis	0/53	1.9%	0/56	5.4%	0.0 (-6.8–6.6)
Bronchiolitis	0/53	1.9%	0/56	5.4%	0.0 (-6.8–6.6)
Disseminated intravascular coagulation (DIC)	0/53	0.0%	0/56	5.4%	0.0 (-6.8–6.8)
Other cardiac arrhythmia	1/53	0.0%	1/56	7.1%	0.0 (-9.8–9.6)
Hypoglycemia	0/53	1.9%	1/56	5.4%	-1.9 (-9.9–6.3)

Pancreatitis	0/53	1.9%	1/56	5.4%	-1.9 (-9.9–6.3)
Stroke	0/53	0.0%	1/56	5.4%	-1.9 (-9.9–6.4)
Cryptogenic organizing pneumonia (COP)	0/53	1.9%	1/56	5.4%	-1.9 (-9.9–6.3)
New atrial fibrillation or flutter (AF)	1/53	1.9%	2/56	7.1%	-2.0 (-12.7–8.7)
Pulmonary embolism (PE)	6/53	1.9%	7/56	5.4%	-2.1 (-19.6–15.6)
Pleural effusion	8/53	1.9%	9/56	5.4%	-2.2 (-21.5–17.4)
Decompensated heart failure	0/53	0.0%	2/56	7.1%	-3.8 (-13.0–5.7)
Meningitis or encephalitis	0/53	1.9%	2/56	5.4%	-3.8 (-12.8–5.6)
Acute Respiratory Distress Syndrome (ARDS)	12/53	1.9%	14/56	5.4%	-4.2 (-26.4–18.5)
Non-ST-elevation myocardial infarction (NSTEMI)	0/53	1.9%	3/56	5.4%	-5.7 (-15.4–4.7)
Pneumothorax	2/53	1.9%	6/56	5.4%	-7.6 (-21.6–7.2)
Other complications	34/53	1.9%	39/56	5.4%	-10.6 (-33.9–14.2)
Bacteremia	3/53	0.0%	9/56	5.4%	-11.3 (-27.3–6.2)

Figure S6. Variant differences in in-hospital conditions (unadjusted risk differences with 95% CIs).
Shown are the top five conditions by absolute risk difference (RD) comparing Delta and Omicron. **RD = P(condition | Delta) − P(condition | Omicron),** expressed in percentage points; the dot is the point estimate, and the horizontal bar is the 95% CI. The vertical dashed line (RD=0) marks no between-variant difference. Analyses are exploratory and unadjusted. **See Supplementary Table S6** for the full counts, missingness, and RD 95% CIs for all conditions.

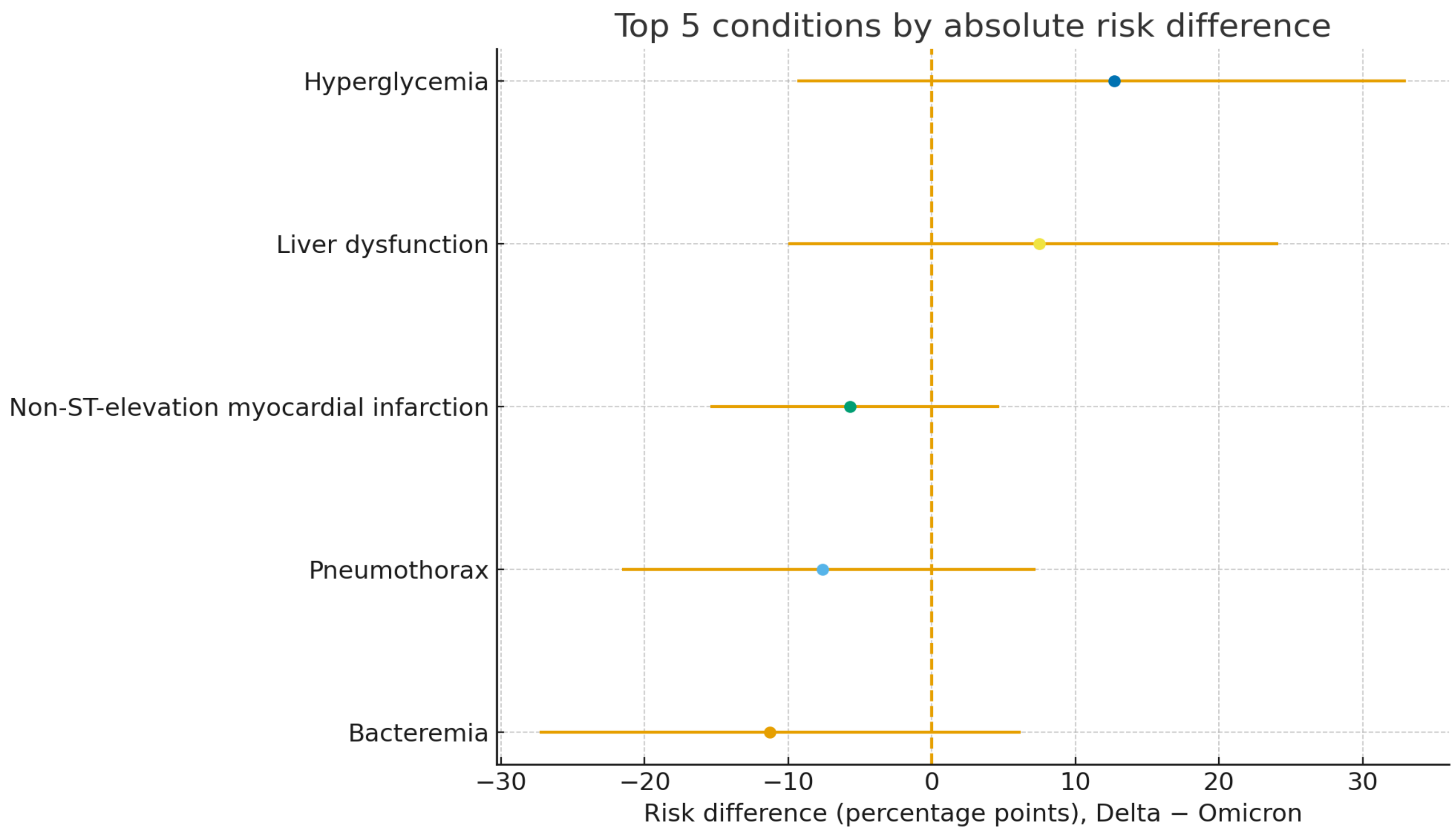

Table S7. Cox model for mortality: Delta vs Omicron with baseline covariates. Outcome = time from admission to in-hospital death; discharges censored at length of stay. Model reports hazard ratios (HR) with 95% CI and *p*-values; HR>1 indicates higher hazard. Covariates: variant (Delta vs Omicron), NIH severity (ordinal 1–4), age (per year), male vs female, vaccinated (any dose vs none). Estimation by Cox PH with Efron ties; complete-case analysis due to missing vaccination. Abbrev: **HR**, hazard ratio; **CI**, confidence interval.

	coef	HR	HR 95% CI low	HR 95% CI high	p-value
**Variant: Delta vs Omicron**	1.848	6.346	2.222	18.129	0.001
**Severity (ordinal 1–4)**	0.990	2.691	1.398	5.180	0.003
**Age (per year)**	0.043	1.044	1.011	1.078	0.008
**Male vs Female**	-0.489	0.614	0.256	1.472	0.274
**Vaccinated (any dose)**	-0.989	0.372	0.154	0.896	0.028

REFERENCES

1. Jin J, Byun JK, Choi YK, Park KG. Targeting glutamine metabolism as a therapeutic strategy for cancer. Exp Mol Med. 2023;55(4):706-15.
2. Deelen J, Kettunen J, Fischer K, van der Spek A, Trompet S, Kastenmüller G, et al. A metabolic profile of all-cause mortality risk identified in an observational study of 44,168 individuals. Nat Commun. 2019;10(1):3346.
3. Boccard J, Veuthey JL, Rudaz S. Knowledge discovery in metabolomics: An overview of MS data handling. Journal of Separation Science. 2010;33(3):290-304.
4. Hardikar S, Albrechtsen RD, Achaintre D, Lin T, Pauleck S, Playdon M, et al. Impact of Pre-blood Collection Factors on Plasma Metabolomic Profiles. Metabolites. 2020;10(5).
5. Bordag N, Klie S, Jürchott K, Vierheller J, Schiewe H, Albrecht V, et al. Glucocorticoid (dexamethasone)-induced metabolome changes in healthy males suggest prediction of response and side effects. Sci Rep. 2015;5:15954.
6. Newgard CB, An J, Bain JR, Muehlbauer MJ, Stevens RD, Lien LF, et al. A branched-chain amino acid-related metabolic signature that differentiates obese and lean humans and contributes to insulin resistance. Cell Metab. 2009;9(4):311-26.
7. Kalim S, Rhee EP. An overview of renal metabolomics. Kidney Int. 2017;91(1):61-9.
8. Godbole S, Bowler RP. Metabolome Features of COPD: A Scoping Review. Metabolites. 2022;12(7).
9. Cevik M, Tate M, Lloyd O, Maraolo AE, Schafers J, Ho A. SARS-CoV-2, SARS-CoV, and MERS-CoV viral load dynamics, duration of viral shedding, and infectiousness: a systematic review and meta-analysis. Lancet Microbe. 2021;2(1):e13-e22.
10. Chen Z, Fung E, Wong CK, Ling L, Lui G, Lai CKC, et al. Early Metabolomic and Immunologic Biomarkers as Prognostic Indicators for COVID-19. Metabolites. 2024;14(7).
